# A Solution Method for Non-Linear Underdetermined Equation Systems in Grounding Grid Corrosion Diagnosis Based on an Enhanced Hippopotamus Optimization Algorithm

**DOI:** 10.3390/biomimetics10070467

**Published:** 2025-07-16

**Authors:** Jinhe Chen, Jianyu Qi, Yiyang Ao, Keying Wang, Xin Song

**Affiliations:** 1 Tianyou College, East China Jiaotong University, Nanchang 330000, China; jinhechen666@163.com (J.C.); 18992516243@163.com (J.Q.); aoyiyang0529@163.com (Y.A.); 2 School of Natural Sciences, University of Manchester, Manchester M13 9PL, UK; keying.wang@student.manchester.ac.uk; 3 School of Sciences, East China Jiaotong University, Nanchang 330000, China

**Keywords:** grounding grid corrosion diagnostics, nonlinear under-determined systems of equations, Enhanced Hippopotamus Optimization (EBOHO), beta-function population initialization, elite–mean search operator, lens imaging opposition-based learning

## Abstract

As power grids scale and aging assets edge toward obsolescence, grounding grid corrosion has become a critical vulnerability. Conventional diagnosis must fit high-dimensional electrical data to a physical model, typically yielding a nonlinear under-determined system fraught with computational burden and uncertainty. We propose the Enhanced Biomimetic Hippopotamus Optimization (EBOHO) algorithm, which distills the river-dwelling hippo’s ecological wisdom into three synergistic strategies: a beta-function herd seeding that replicates the genetic diversity of juvenile hippos diffusing through wetlands, an elite–mean cooperative foraging rule that echoes the way dominant bulls steer the herd toward nutrient-rich pastures, and a lens imaging opposition maneuver inspired by moonlit water reflections that spawn mirror candidates to avert premature convergence. Benchmarks on the CEC 2017 suite and four classical design problems show EBOHO’s superior global search, robustness, and convergence speed over numerous state-of-the-art meta-heuristics, including prior hippo variants. An industrial case study on grounding grid corrosion further confirms that EBOHO swiftly resolves the under-determined equations and pinpoints corrosion sites with high precision, underscoring its promise as a nature-inspired diagnostic engine for aging power system infrastructure.

## 1. Introduction

The grounding network is a critical component of the power system, primarily serving to provide safe and reliable grounding protection for electrical equipment, thereby ensuring the stable operation of the power system. The performance of the grounding network is directly linked to the safety and operational reliability of electrical equipment; consequently, its condition monitoring and fault diagnosis are of paramount importance in the operation and maintenance of the power system [1]. However, over extended periods of service, many grounding networks—particularly those installed in the 1980s and 1990s—have undergone significant aging and are increasingly subjected to complex environmental and operational stressors, including humidity, temperature fluctuations, soil corrosivity, and stray direct currents introduced by high-voltage direct current (HVDC) systems [2]. These factors collectively accelerate corrosion, significantly degrade the network’s conductivity, and may even lead to grounding failure. Such degradation compromises protective relay sensitivity, alters fault current dissipation paths, and poses risks to both system stability and personnel safety. Therefore, the timely and accurate diagnosis of grounding network corrosion has become an increasingly critical task for ensuring the safety and reliability of modern power systems [3].

Currently, research on grounding network corrosion diagnosis primarily employs electromagnetic, electrochemical, and electrical network methods. The electromagnetic method infers the state of the grounding network by measuring the responses of current and voltage; however, due to its poor adaptability to the internal structure of the grounding network and its inability to accurately reflect the specific locations of corrosion, its application is limited in complex grounding network systems [4,5,6]. The electrochemical method, on the other hand, assesses the degree of corrosion by measuring the electrochemical properties of the grounding network. While this method can directly reflect the corrosion process, its limitations include the complexity of equipment installation, the cumbersome nature of the experimental process, and its susceptibility to environmental factors, making it challenging to apply in the real-time monitoring of large-scale grounding networks [7,8].

In contrast, the electrical network method offers a modeling approach based on the electrical characteristics of the grounding network [9]. By constructing an electrical network model of the grounding network, it is possible to simulate its electrical behavior and reflect the impact of corrosion on the network’s electrical performance. The electrical network method can flexibly describe the structural characteristics of the grounding network and solve it effectively using circuit analysis techniques, making it suitable for various complex grounding network systems. Consequently, the electrical network method holds distinct advantages in grounding network corrosion diagnosis, particularly when dealing with large-scale, intricately structured networks [10]. However, corrosion diagnostic models based on the electrical network method are typically converted into underdetermined and highly ill-conditioned nonlinear systems of equations, which may possess multiple possible solutions, presenting significant challenges in solving the problem. If these under-determined models are not solved accurately, corrosion hot spots can be overlooked, leading to unexpected rises in grid-to-ground potential and protective relay maloperations in live substations. Traditional analytical methods and gradient optimization approaches often face difficulties, such as excessively high computational complexity and poor convergence to the global optimum, when addressing such problems [11]. Yet most existing meta-heuristics are configured for fully determined benchmark problems and tend to suffer premature convergence or prohibitive runtime when faced with the highly ill-conditioned, sparsity-constrained solutions required here. The rise of high-dimensional electrical data further compounds these challenges, as the number of accessible nodes in real-world systems is often limited. This high-dimensionality amplifies the underdetermined nature of the system, increasing both the computational burden and the susceptibility to measurement errors. The complexity of finding a reliable solution becomes even greater as dimensionality increases, making it difficult for traditional methods to achieve accurate results. Therefore, there is an urgent need to develop and propose efficient and robust optimization algorithms to resolve the complex solving challenges in grounding network corrosion diagnosis.

**Figure 1:**
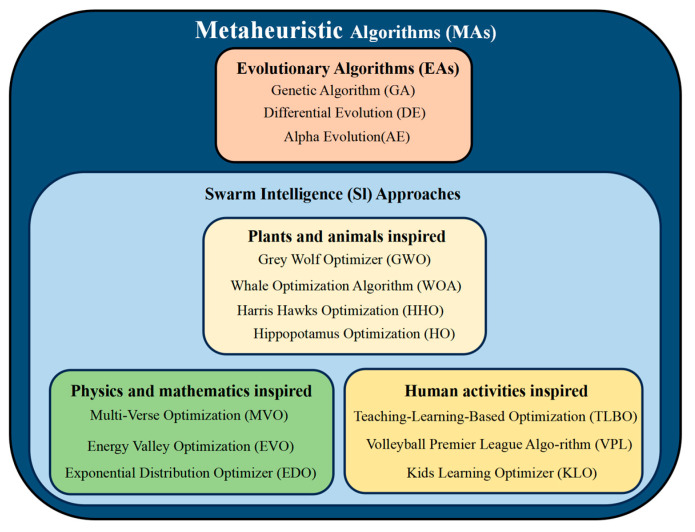
Classification of representative MAs.

In recent years, metaheuristic algorithms [12,13,14], with their powerful global optimization capabilities, have been widely applied to solve complex optimization problems. These algorithms, by simulating natural phenomena, biological behaviors, or physical processes, effectively explore the solution space and seek global optima. This is especially relevant to the grounding grid corrosion diagnosis problem investigated in this study, which leads to complex, underdetermined, and highly nonlinear systems of equations. Such characteristics make it difficult for traditional analytical or gradient-based approaches to yield stable solutions. Metaheuristic algorithms, due to their global search capabilities and robustness to problem ill-conditioning, are well suited to tackle this class of inverse diagnostic problems. Based on their sources of inspiration, metaheuristic algorithms can be categorized into four major types: (1) Evolutionary Algorithms: These algorithms, inspired by natural selection and species evolution, achieve a balance between global search and local development through mechanisms such as genetic processes, mutation, and selection. Representative algorithms include the Genetic Algorithm (GA) [15], Differential Evolution (DE) [16], the Alpha Evolutionary Algorithm (AE) [17], and Particle Swarm Optimization (PSO) [18]. (2) Algorithms Inspired by Animal and Plant Populations: These algorithms simulate the cooperative and competitive behaviors of animal and plant populations, demonstrating excellent global optimization capabilities. Typical algorithms in this category include Grey Wolf Optimization (GWO) [19], the Whale Optimization Algorithm (WOA) [20], Harris Hawks Optimization (HHO) [21], Hippopotamus Optimization (HO) [22], and the Crawfish Optimization Algorithm (COA) [23]. (3) Algorithms Based on Physical or Mathematical Principles: These algorithms perform optimization by simulating physical phenomena or mathematical laws. Representative algorithms include Multi-Verse Optimization (MVO) [24], Energy Valley Optimization (EVO) [25], Weighted Mean of Vectors Optimization (INFO) [26], and Exponential Distribution Optimization (EDO) [27]. (4) Algorithms Inspired by Human Activities: These algorithms simulate human behaviors or societal activities and draw upon human experiences to perform search and optimization. Notable algorithms include Teaching–Learning-Based Optimization (TLBO) [28], the Volleyball Premier League Algorithm (VPL) [29], the Alibaba and the Forty Thieves Algorithm (AFT) [30], and the Children Learning Optimization Algorithm (KLO) [31]. Figure 1 illustrates some of the algorithmic categorization details. These metaheuristic algorithms, through strategies such as dynamic adaptation, information sharing, and balancing local and global searches, have demonstrated broad applicability and strong optimization capabilities across multiple fields, making them particularly suited for solving high-dimensional, complex optimization problems.

Hippopotamus Optimization (HO), as an emerging metaheuristic optimization method, has attracted increasing attention from researchers in recent years due to its flexible exploration and exploitation capabilities. However, HO still faces several challenges when solving complex optimization problems, such as a propensity to become trapped in local optima, insufficient population diversity, and slower convergence in the later stages. To address these limitations, scholars have proposed various modified versions of HO to further enhance its global search performance and solving efficiency. For instance, Tao Han and colleagues introduced the Modified Hippopotamus Optimization (MHO) algorithm, an improved version of the HO, to solve global optimization and engineering design problems [32]. Building upon HO, S. Punitha and colleagues, inspired by scrolls, proposed the Scroll-Inspired Hippopotamus (SIH) algorithm to generate the most random optimum keys [33]; Hongbin Wang et al. proposed the IHO algorithm, which uses Latin Hypercube Sampling (LHS) for population initialization and integrates the Jaya algorithm to improve solution quality and convergence speed. The optimization process is enhanced with unordered dimension sampling, random crossover, and sequential mutation. The effectiveness of IHO is demonstrated in predicting solar photovoltaic power generation by optimizing weights and neuron thresholds in an Extreme Learning Machine (ELM) [34]. Yuxin He et al. proposed GAHO, which enhances the Hippopotamus Optimization (HO) algorithm by integrating non-uniform Gaussian mutation, Genetic Algorithm (GA)-based individual crossover, and phase parameter adjustment. These modifications significantly improve the algorithm’s global optimization ability and convergence speed, and they have been applied to the design of FOPID controllers for pneumatic control valves [35]. However, most of these improvements are developed for general optimization tasks and lack adaptation to the nonlinear underdetermined systems of equations encountered in grounding network corrosion diagnosis, especially in high-dimensional settings. Moreover, several promising strategies, including β-distribution-based initialization, elite-to-mean cooperative operators, and lens imaging-based opposition learning, have not yet been integrated into a unified HO framework, leaving their combined effectiveness unexplored.

This study aims to develop a customized and robust metaheuristic optimization method for solving underdetermined nonlinear equation systems, with a focus on grounding network corrosion diagnosis. To address these issues, we propose a multi-strategy enhanced HO algorithm (EBOHO). The main contributions of this paper are as follows:

(1) The population is initialized using a beta function distribution, which enhances the diversity and uniformity of the population. This initialization method effectively avoids the issue of population concentration that may arise from traditional random initialization, providing a broader exploration of the solution space and improving the coverage of the initial search.
(2) By combining the current optimal solution with the mean information of the population, an elite–mean operator is introduced, which strengthens the balance between global exploration and local exploitation during the search process. This operator accelerates the algorithm’s convergence while maintaining population diversity, effectively avoiding local optima and enhancing both the global search capability and convergence efficiency.
(3) A lens imaging reverse learning strategy is introduced. This strategy simulates the lens imaging process to dynamically adjust the position of solutions, enabling individuals to escape local optima and expand the search range. This helps prevent premature convergence to local optima, improves the breadth of the search, enhances global search capabilities, and ensures that the algorithm fully explores the potential of the solution space during optimization.
(4) The algorithm is experimentally evaluated on the CEC2017 benchmark test set in various dimensions, as well as on four classic engineering design problems. It is compared with multiple optimization algorithms, and the superiority of the algorithm is further validated using the Mann–Whitney U test and Friedman ranking.
(5) EBOHO is applied to the grounding network corrosion diagnosis problem to solve complex underdetermined systems of equations. The experimental results demonstrate the effectiveness and robustness of EBOHO, confirming its advantages and practical application potential in optimization problems.

The remainder of this paper is organized as follows: Section 2 introduces the mathematical model of HO, Section 3 provides a detailed description of the proposed improved HO algorithm, Section 4 focuses on CEC numerical experiments and statistical analysis, Section 5 presents experiments on and analysis of classical engineering design problems, and Section 6 demonstrates the superiority of the algorithm in solving underdetermined problems through its application to the grounding network corrosion diagnosis model. Finally, Section 7 concludes the paper and outlines several open topics for future research.

## 2. Basic Principles of the Hippopotamus Optimization (HO) Algorithm

The Hippopotamus Optimization (HO) algorithm, proposed by Mohammad Hussein Amiri et al. [22] in February 2024, is inspired by the behavior of hippopotamuses. The algorithm primarily includes the initial positions of the hippo population, their position updates within the water environment, their defense mechanisms against predators, and their strategies for escaping predators. By adaptively adjusting the resolution and search speed of the search space, the HO algorithm is able to efficiently and accurately find the optimal solution.

(1) Initialization of the Hippo Population

Similar to traditional swarm intelligence optimization algorithms, the initialization process of this algorithm also follows a random initialization approach, where the initial solutions are randomly generated. The specific formula is as follows:

(1) $$X_{i,j}=Y_{l,j}+r(Y_{u,j}-Y_{l,j})\begin{array}{cc} & i=1,2,\ldots N;\;j=1,2,\ldots M \end{array}$$

where N is the population size, M is the number of decision variables for the problem, $X_{i,j}$ represents the candidate position of the i-th solution, and j indicates the current decision variable. r is a random number in the range of [0,1], and $Y_{u,j}$ and $Y_{l,j}$ are the lower and upper bounds of the j-th decision variable, respectively.

(2) Position Update of Hippos in the Lake or Pond

The hippo population consists of adult female hippos, young hippos, several adult male hippos, and a dominant male hippo (the leader). In the hippo population, the status of male hippos is crucial. The dominant male hippo protects the group and territory from threats, while female hippos typically gather around the male hippos. As male hippos mature, they are often displaced by higher-ranking males within the group. The displaced male hippos face a choice: either attract female hippos or compete with other male hippos in the group to vie for dominance. The changes in the positions of male hippos within the group can be described by Equations (2) and (3), which reflect the complex interactions and position updates among male hippos in the group.

(2) $$X_{i,j}^{Mhippo}=X_{i,j}+r(X^{Dhippo}-I_{1}X_{i,j})\begin{array}{cc} & \;i=1\;,2,\ldots N/2;\;j=1,2,\ldots M \end{array}$$

(3) $$S=\left\{\begin{array}{l} I_{2}\times r_{1}+(\sim p_{1})\\ 2\times r_{2}-1\\ r_{3}\\ I_{1}\times r_{4}+(\sim p_{2})\\ r_{5} \end{array}\right\}$$

Here, $X_{i,j}^{Mhippo}$ represents the position of the male hippo, $X_{i,j}^{Dhippo}$ is the position of the best hippo in the current iteration, S refers to five randomly selected scenarios, $I_{1},I_{2}$ is an integer within the range of $\left[1,2\right]$, $\sim p_{1},\sim p_{2}$ is an integer random number in the range of $\left[0,1\right]$, $r_{1}\sim r_{4}$ is a random vector within the range of $\left[0,1\right]$, and $r_{5}$ is a random number within the range of $\left[0,1\right]$.

The positions of female or immature hippos within the population are described in Equations (4)–(6). Most immature hippos stay close to their mothers, but due to curiosity, they may sometimes leave the group. If T>0.6, it indicates that the immature hippo has moved away from the mother. If, at this point, the random number $r_{6}>0.5$, it indicates that the immature hippo, although separated from the group, remains nearby. Otherwise, it has fully departed from the group.

(4) $$T=\exp(-\frac{t}{T_{\max}})$$

(5) $$X_{i,j}^{FBhippo}=\left\{\begin{array}{l} X_{i,j}+S_{1}(X^{Dhippo}-I_{2}h_{i})T>0.6\\ Z\begin{array}{cc} & \;\;\;\;else \end{array} \end{array}\right\}$$

(6) $$Z=\left\{\begin{array}{l} X_{i,j}+S_{2}(h_{i}-X_{i,j}^{Dhippo})r_{6}>0.5\\ Y_{l,j}+r_{7}(Y_{u,j}-Y_{l,j})else \end{array}\right\}$$

Here, T represents the distance between the current iteration number and the maximum iteration number, t is the current iteration number, and $T_{max}$ is the maximum iteration number. $X_{i,j}^{FBhippo}$ denotes the position of the immature hippo, $h_{i}$ is the average position of some randomly selected hippos, $S_{1},S_{2}$ represents the state of the five randomly selected scenarios in Equation (S), Z indicates the status of the immature hippo’s departure from the group, including whether it is near the population or has fully left the group, and $r_{7}$ is a random number in the range of $\left[0,1\right]$. Based on Equation (6), the position update formulas for the male, female, or immature hippos in the population are as follows:

(7) $$X_{i}=\left\{\begin{array}{cc} X_{i}^{Mhippo} & F_{i}^{Mhippo}<F_{i}\\ X_{i} & else \end{array}\right\}$$

(8) $$X_{i}=\left\{\begin{array}{cc} X_{i}^{FBhippo} & F_{i}^{FBhippo}<F_{i}\\ X_{i} & else \end{array}\right\}$$

where $F_{i}$ represents the objective function value, $F_{i}^{Mhippo}$ is the objective value of the male hippo, and $F_{i}^{FBhippo}$ is the objective value of the female or immature hippo.

(3) Hippo Defense Exploration Phase

Hippopotamuses, due to their large size, can effectively prevent predators from approaching. However, due to curiosity, immature hippos may leave the group, making them vulnerable to predators such as lions and hyenas. Compared to adult hippos, they are weaker, and with the potential presence of sick individuals in the group, these hippos are more easily targeted by predators. To protect themselves from the threat of predation, hippos typically adopt defensive behaviors, such as quickly turning and emitting loud calls to intimidate predators and prevent them from getting closer. The current position of the predator is considered part of the search area.

(9) $$X_{j}^{\mathrm{Pr}edator}=Y_{l,j}+r_{8}(Y_{u,j}-Y_{l,j})\begin{array}{cc} & j=1,2\ldots M \end{array}$$

(10) $$D=\left|X_{j}^{\mathrm{Pr}edator}-X_{i,j}\right|$$

Here, $r_{8}$ is a random vector in the range of $\left[0,1\right]$, and D is the distance between the hippo and the predator. During this process, the hippo employs a defense mechanism determined by factor $F_{j}^{\mathrm{Pr}edator}$. $F_{j}^{\mathrm{Pr}edator}$ represents the objective value of the hippo during the defense exploration phase. If $F_{j}^{\mathrm{Pr}edator}$ is less than $F_{i}$, it indicates that the predator is very close, prompting the hippo to quickly turn towards the predator and approach it in order to make it retreat. If $F_{j}^{\mathrm{Pr}edator}$ continues to increase, it suggests that the predator is farther away, so the hippo turns towards the predator with a lower level of aggression, as described in Equation (11).

(11) $$X_{i,j}^{HippoR}=\left\{\begin{array}{cc} RL⊕X_{j}^{\mathrm{Pr}edator}+\frac{\alpha }{c-d\times \cos(2\pi \beta )}\times \frac{1}{D} & F_{j}^{\mathrm{Pr}edator}<F_{i}\\ RL⊕X_{j}^{\mathrm{Pr}edator}+\frac{\alpha }{c-d\times \cos(2\pi \beta )}\times \frac{1}{2\times D+r_{9}} & F_{j}^{\mathrm{Pr}edator}\geq F_{i} \end{array}\right\}$$

Here, $X_{i,j}^{HippoR}$ represents the posture of the hippo facing the predator, and ⊕ is the result of an XOR operation. Here, ⊕ denotes a binary selection operation: each dimension is randomly chosen from either RL or $X_{j}^{\mathrm{Pr}edator}$, controlled by a binary decision mask, $\theta \in {\left\{0,1\right\}}^{D}$. α is a uniformly distributed random number within the range of $\left[2,4\right]$, c is a uniformly distributed random number within the range of $\left[1,1.5\right]$, d is a uniformly distributed random number within the range of $\;\left[2,3\right]$, β is a random number within the range of $\left[-1,1\right]$, $r_{9}$ is a random vector of 1×M, and RL is a random vector with a Levy distribution that is used to represent the position mutation of the predator when the hippo is attacked. The mathematical model of the Levy distribution is as follows:

(12) $$Levy(\vartheta )=0.05\times \frac{\omega \times \sigma_{\omega }}{{\left|\mu \right|}^{\frac{1}{\theta }}}$$

(13) $$\sigma_{\omega }={\left[\frac{\Gamma (1+\vartheta )\sin(\frac{\pi \vartheta }{2})}{\Gamma \frac{1+\vartheta }{2}\vartheta \times 2^{\frac{\vartheta -1}{2}}}\right]}^{\frac{1}{\vartheta }}$$

In the equation, ω and μ are random numbers within the range of $\left[0,1\right]$, ϑ is a constant equal to 1.5, $\sigma_{\omega }$ represents the Levy step size, and Γ is the Gamma function.

(14) $$X_{i}=\left\{\begin{array}{cc} X_{i}^{HippoR} & F_{i}^{HippoR}<F_{i}\\ X_{i} & F_{i}^{HippoR}\geq F_{i} \end{array}\right\}$$

Equation (14) is the final position of the hippopotamus at that stage, and $F_{i}^{HippoR}$ is the target value of the hippopotamus at the final position. When $F_{i}^{HippoR}\geq F_{i}$, it indicates that the hippopotamus has been killed and replaced by another hippopotamus, and when the opposite is true, the predator escapes and the hippopotamus returns to the population.

(4) Hippo Escape from Predator Exploitation Phase

When faced with multiple predators or unsuccessful defense attempts to ward them off, hippos adopt a retreat strategy. They typically seek refuge by swiftly moving toward the nearest body of water. This behavior is part of the third phase of the HO model, enhancing their ability to effectively explore nearby safe zones. When the newly created position increases the cost function value, it indicates that the hippo has found a safer location near its current position and has relocated to this safe place.

(15) $$\left\{\begin{array}{c} Y_{i,j}^{local}=\frac{Y_{i,j}}{t}\\ Y_{u,j}^{local}=\frac{Y_{u,j}}{t} \end{array}\right\}\begin{array}{cc} & t=1,2,\ldots ,T_{max} \end{array}$$

(16) $$X_{i,j}^{Hippo\varepsilon }=X_{i,j}+r_{10}[Y_{l,j}^{local}+K(Y_{u,j}^{local}-Y_{l,j}^{local})]$$

Here, $Y_{u,j}^{local},Y_{i,j}^{local}$ represent the upper and lower bounds of the current safe position, respectively, $r_{10}$ is a random number in the range of $\;\left[0,1\right]$, $X_{i,j}^{Hippo\varepsilon }$ is the position of the hippo searching for the nearest safe location, and K is randomly selected from three scenarios described by the following formula. These scenarios influence more optimal local searches, enhancing the algorithm’s ability to exploit higher-quality solutions.

(17) $$K=\left\{\begin{array}{c} 2\times r_{11}-1\\ r_{12}\\ r_{3} \end{array}\right\}$$

In the equation, $r_{11}$ is a random vector within the range of $\left[0,1\right]$, $r_{12}$ is a random number that follows a normal distribution, and $r_{13}$ is a random number within the range of $\left[0,1\right]$.

Finally, the position of the optimal population is updated, as shown in the following equation:

(18) $$X_{i}=\left\{\begin{array}{cc} X_{i}^{Hippo\varepsilon } & F_{i}^{Hippo\varepsilon }<F_{i}\\ X_{i} & F_{i}^{Hippo\varepsilon }\geq F_{i} \end{array}\right\}$$

where $F_{i}^{Hippo\varepsilon }$ is the objective value that corresponds to the position of the optimal population.

## 3. The Proposed EBOHO Algorithm

The traditional Hippopotamus Optimization (HO) algorithm has several notable shortcomings, including a lack of diversity during population initialization, a tendency to get trapped in local optima, and the potential for premature convergence during the search process. To overcome these issues, we propose the EBOHO algorithm, which introduces three key strategies. First, beta function-based population initialization is employed to enhance the diversity and uniformity of the population, providing a more robust starting point for global search. Second, by introducing the elite–mean operator, which combines the current best solution with the population mean, the balance between global exploration and local exploitation is strengthened, effectively accelerating convergence and preventing premature convergence to local optima. Finally, the lens imaging reverse learning strategy is adopted, which helps individuals escape local optima, thereby broadening the search range and improving global search capabilities.

### 3.1. Beta Function Initialization (Beta)

The optimization process is essentially the search for the optimal solution within the solution space. The advantage of swarm intelligence optimization lies in the collaborative effort of population members, who work together to approach the optimal solution, effectively ‘besieging’ the optimal solution. Therefore, the setting of the initial population positions significantly influences the search process. The traditional HO algorithm uses a simple and straightforward method of randomly generating initial positions, but this approach is not conducive to ‘surrounding’ the optimal solution. To enable the initial positions to effectively encircle the optimal solution, we propose a population initialization method based on the beta distribution.

The definition of the beta distribution function is as follows:

(19) $$\beta (x)=\frac{x^{a-1}{\left(1-x\right)}^{b-1}}{B(a,b)},0<x<1$$

where the denominator is the β function, which is defined as follows:

(20) $$B(a,b)=\int_{0}^{1}t^{a-1}{\left(1-t\right)}^{b-1}dt$$

where a=b<1.

**Figure 2:**
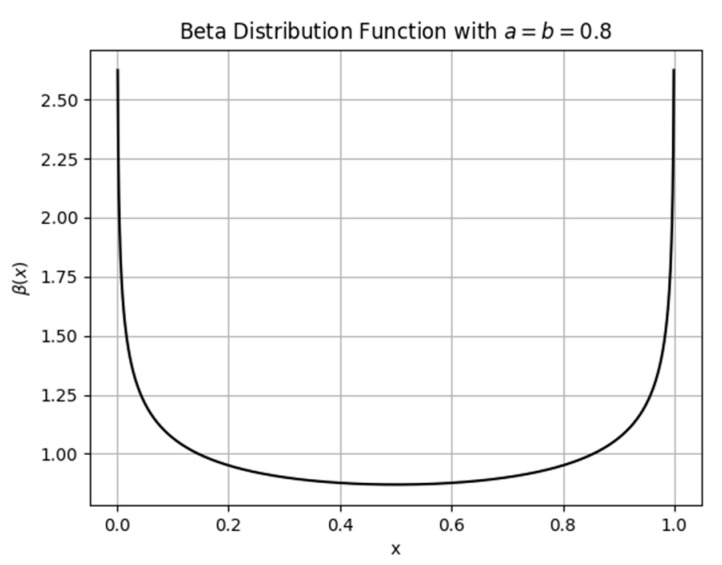
Beta distribution function, where a = b = 0.8.

In this case, the shape of the density function is a symmetric U shape. As a result, the candidate solutions are most likely to be located near the boundaries of the search space, effectively ‘surrounding’ the global optimum within the initial particle population. Extensive experiments have shown that when a=b=0.8, the initialization performs well. This parameter setting is also supported by several metaheuristic studies. Li et al. (2020) [36] and Agushaka and Ezugwu (2022) [37] found that symmetric beta initializations with parameters within the range of 0.5 to 3 significantly improve population diversity and convergence behavior in PSO, DE, and other optimizers. Specifically, a = b = 0.8 was used and recommended in Jian et al. (2017) [38] as an effective default in swarm-based algorithms. Although no further parameter tuning is performed in this study, the chosen value is already widely adopted and empirically validated, thus offering a robust and literature-supported trade-off without the need for exhaustive sensitivity testing. The probability density function of this distribution over the interval (0, 1) is shown in Figure 2. Based on the β probability distribution, initial values for each dimension of $X_{i}$ are assigned according to the following formula:

(21) $$X_{i,j}=Y_{l,j}+(Y_{u,j}-Y_{l,j})\times betarnd(a,b,1,1)$$

In the traditional HO initialization method, uniform random numbers are used, resulting in an initialization that is approximately uniform throughout the search space. This is not conducive to surrounding the optimal solution. However, the population initialization method based on the beta distribution overcomes this limitation.

### 3.2. Elite–Mean Operator (EMO)

In the traditional HO algorithm, the search of the population is often constrained by the interactions among individuals, making it prone to getting stuck in local optima, especially when solving complex, high-dimensional problems. To address this issue, we introduce the global optimal solution (i.e., the elite solution) and the mean solution of the population (i.e., the average solution). By combining these two solutions through a weighted sum, new search points are generated. This approach not only ensures the global search capability of the algorithm but also accelerates the convergence process. The core idea of this operator is that the elite solution represents the current optimal exploration direction of the population, while the mean solution represents the overall trend of the population. By combining the elite and mean solutions, the diversity of the population can be maintained, and premature convergence can be avoided. The details are introduced as follows:

First, the mean position of all individuals in the current population is calculated, as shown in Equation (22). The population mean represents the overall ‘center position’ of the current population, reflecting the average distribution of the population members in the solution space. By calculating the mean of the population, a reference point is obtained, serving as an overall reference for the positions of all individuals.

(22) $$Mean=\frac{1}{N}\sum_{i=1}^{N}X_{i}$$

Next, using the current global optimal solution, $X_{best}$, and the mean solution of the population, Mean, we combine both to calculate new search points. The specific weighted combination formula is as follows:

(23) $$ElMean=0.7\cdot X_{best}+0.3\cdot Mean$$

Here, 0.7 and 0.3 are weighting coefficients assigned to the global best solution and the population mean, respectively. This asymmetric combination gives more influence to the best-known solution while still incorporating the population’s overall trend. The chosen ratio, which is commonly used in elite-guided strategies in metaheuristics, such as CMA-ES and the Equilibrium Optimizer, provides a practical trade-off between convergence accuracy and global search capability.

Finally, the positions of the individuals are updated, as shown in Equation (24). The purpose of this formula is to guide the individuals’ positions toward a better direction under the influence of the elite solution and the mean solution, while avoiding over-reliance on a single optimal solution, thus maintaining the diversity and global nature of the search.

(24) $$X_{EM}(i,:)=X(i,:)+r_{14}\cdot (ElMean-X(i,:))$$

Here, $r_{14}$ is a randomly generated coefficient within the range of $\left[0,1\right]$ that is used to adjust the step size at which individuals move toward the elite mean point.

### 3.3. Lens Imaging Reverse Learning (LlRL)

The traditional HO algorithm is prone to getting trapped in local optima, particularly in complex high-dimensional optimization problems, where the issue of local optima is particularly prominent. The Lens Imaging Reverse Learning (LIRL) strategy simulates the process of lens imaging to perform a reverse mapping of the individual’s current position, allowing the individual to escape the current local optimum region and explore other areas of the solution space. The improved algorithm applies Lens Imaging Reverse Learning after each iteration using the following formula:

(25) $$X_{i}^{*}=\frac{Y_{u,j}+Y_{l,j}}{2}+\frac{Y_{u,j}+Y_{l,j}}{2\lambda }-\frac{X_{i}}{\lambda }$$

where $X_{i}^{*}$ represents the reverse solution space of $X_{i}$, and λ is the reverse imaging parameter, which influences the size of the optimization region for reverse learning. Here, $Y_{u,j}$ and $Y_{l,j}$ denote the upper and lower bounds of the decision variable, $X_{j}$, respectively, which is consistent with the notation used in Equation (1).

To facilitate extensive exploration in the early stages of the search and gradual convergence in the later stages, we have designed a dynamic adjustment for the reverse imaging parameter, λ, which controls the intensity of reverse imaging. Initially, λ is set to a larger value to expand the search range, while in the later iterations, λ approaches 1 to promote the convergence process. The specific formula is as follows:

(26) $$\lambda =1+r_{15}(1-\frac{t}{T_{\max}})$$

where $r_{15}$ is a random number within the range of $\;\left[0,1\right]$. As the number of iterations increases, the value of λ gradually decreases, thereby reducing the intensity of reverse imaging and preventing excessive searching.

(27) $$X_{i}=\left\{\begin{array}{cc} X_{i} & F_{i}<F_{i}^{*}\\ X_{i}^{*} & F_{i}\geq F_{i}^{*} \end{array}\right.$$

Here, $F_{i}$ and $F_{i}^{*}$ represent the fitness values of the forward and reverse solution spaces, respectively. By comparing the two fitness values, the better individual is selected as the initial position for the next iteration, which helps the algorithm converge more quickly to the optimal solution.

**Figure 3:**
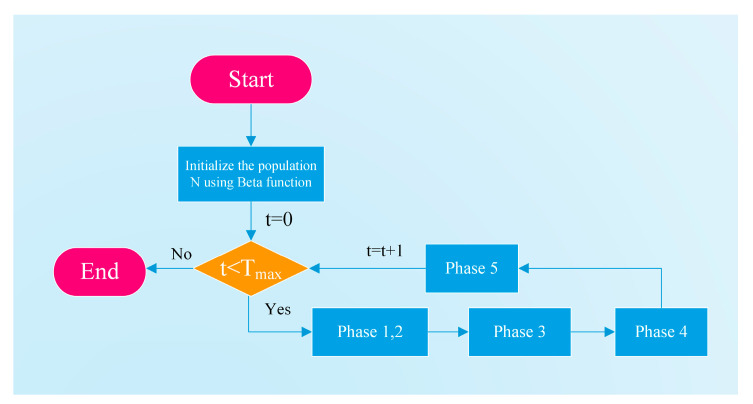
Main flowchart of the EBOHO algorithm. Here, t is the current iteration number, and $T_{\max}$ is the maximum number of iterations. The five phases correspond to the steps defined in Algorithm 1.

Finally, the complete pseudocode of EBOHO is provided in Algorithm 1. Figure 3 simply shows the flowchart of the EBOHO algorithm.
**Algorithm 1.** The pseudocode of EBOHOStart EBOHO1. Define the optimization problem (objective function, variable bounds, and constraints).2. Set the maximum number of iterations, T, and the population size, N (hippopotamuses).3. Generate the initial positions of the N hippos with the beta distribution and evaluate their fitness.4. For t = 1 to T5. Update the dominant hippo (global best) according to fitness.6. //**Phase 1**: Exploration in the river/pond7. For i = 1 to N/28. Compute a new position for hippo i with Equations (2) and (5).9. Update its position using Equations (7) and (8).10. End For11. //**Phase 2**: Defense against predators
12. For i = N/2 + 1 to N13. Generate a random predator position with Equation (9).14. Compute a new position for hippo i using Equation (11).15. Update its position with Equation (14).16. End For17. //**Phase 3**: Exploitation—escaping from the predator18. Recalculate variable bounds using Equation (15).19. For i = 1 to N20. Compute a new position for hippo i with Equation (16).21. Update its position with Equation (18).22. End For23. //**Phase 4**: Elite–mean operator24. Compute the mean position of all agents with Equation (22).25. Form the EliteMeanPoint as a weighted mix of the global best and the mean.26. For i = 1 to N27. Update hippo i via the elite–mean operator (Equation (24)) and accept if fitness improves.28. End For29. //**Phase 5**: Lens imaging reverse learning30. Compute the center of the search space (mid-point of bounds) and adapt λ for exploration size.31. For i = 1 to N32. Update hippo i with the lens imaging strategy, apply boundary check (Equations (25)), accept if fitness improves (Equations (27)).33. End For34. Record the best solution found so far.35. End For36. Output the best overall solution.End EBOHO

### 3.4. Computational Complexity Analysis

Computational complexity measures the number of computational steps required for an algorithm to execute in the worst-case scenario. By analyzing the time complexity, we can assess the algorithm’s operational efficiency and predict its performance for problems of varying sizes. Given that the population size is N, the problem dimension is D, and the maximum number of iterations is T, we will now compare the complexity analysis of the standard HO algorithm and the proposed EBOHO, as detailed below:

The complexity of the standard HO algorithm is primarily composed of the population initialization phase and the position update phase. The time complexity of the initialization phase can be represented as $T_{1}=O(N\times D)$. In the position update phase, hippos use a position update mechanism to defend and escape predators in rivers or ponds, with a complexity of O(N×D) for each iteration. After T iterations, the total time complexity for this part is $T_{2}=O(T\times N\times D)$. Therefore, the overall complexity of the HO algorithm can be expressed as $T_{HO}=T_{1}+T_{2}=O(T\times N\times D)$.

The proposed EBOHO algorithm employs a beta function distribution strategy in the initialization phase, with a time complexity still represented as $T_{3}=O(N\times D)$. In the position update phase, the introduction of the elite–mean operator and Lens Imaging Reverse Learning results in a time complexity of O(N×D) for each iteration. After T iterations, the total time complexity is represented as $T_{4}=O(T\times N\times D)$, and the overall complexity of the algorithm is $T_{EBOHO}=T_{3}+T_{4}=O(T\times N\times D)$. Additionally, Table 1 presents the time complexity results for several common classical algorithms. It is evident that the time complexity of our proposed EBOHO algorithm is largely consistent with these algorithms. To further support this conclusion, we recorded the actual runtime of EBOHO and several baseline algorithms using the same computational platform and experimental parameters. The results on the CEC2017 test suite at 30 and 50 dimensions are presented in Table A6 and Table A7 (see Appendix B). As shown, the execution time of EBOHO is very close to that of the original HO algorithm, confirming that the proposed strategies do not introduce significant computational overhead.

However, thanks to the introduction of three innovative strategies, our algorithm demonstrates greater robustness and faster convergence when solving complex optimization problems. These innovative strategies undoubtedly provide significant support for the optimization and practical application of the algorithm.

## 4. Numerical Experiments on the CEC Benchmark

In this section, we conduct numerical experiments on the CEC benchmark to evaluate the performance of EBOHO. Section 4.1 introduces the experimental setup and environment, Section 4.2 presents the benchmark tests, competitor algorithms, and parameter settings, Section 4.3 summarizes the experimental results from the CEC 2017 tests, and finally, Section 4.4 presents the content of the ablation experiments.

### 4.1. Experimental Setup and Environment

All numerical experiments were conducted under the same software and hardware conditions to ensure a fair comparison of algorithmic performance. The experimental setup was standardized to eliminate external influences, ensuring the reproducibility and reliability of the results.

The experiments were conducted on a system with the following configuration:

- Operating System: Windows 11;
- Processor: AMD Ryzen 7 5800H with Radeon Graphics;
- Memory: 16GB RAM;
- Programming Language: MATLAB R2022b.

By maintaining a consistent computational environment, potential biases introduced by hardware or software variations are minimized, ensuring a fair and objective comparison of the optimization algorithms. We paid particular attention to the uniformity of the experimental environment to ensure the high reliability of the results and provide a stable and reliable foundation for future research. This rigorous experimental design offers a solid foundation for comparing algorithm performance and highlights the superiority and innovation of the proposed algorithm.

### 4.2. Benchmark Functions, Competitor Algorithms, and Parameter Settings

To comprehensively evaluate the overall performance of the proposed EBOHO algorithm, we selected the CEC 2017 benchmark test suite (proposed by the IEEE Congress on Evolutionary Computation), which covers a variety of complex, multi-modal, and high-dimensional optimization problems. Detailed information about this benchmark function set can be found in Appendix A. Additionally, we selected 10 representative optimizers from different categories as competitors to assess the competitiveness of our proposed EBOHO. These competitors can be divided into four categories:

Classic Algorithms: the Genetic Algorithm (GA) and Particle Swarm Optimization (PSO);

High Citation Algorithms: the Sparrow Search Algorithm (SSA) [39], Harris Hawks Optimization (HHO), and the Rime Ice Optimization Algorithm (RIME) [40];

Latest Algorithms: the Starfish Optimization Algorithm (SFOA) [41] and Crested Porcupine Optimization (CPO) [42];

HO Algorithm Variants: HO, SIH, and MHO.

Table 2 summarizes the parameter settings for all competitor algorithms. During the experiments, we set the population size to 100, the maximum number of iterations to 30, and the maximum number of fitness evaluations to 1000×D.

### 4.3. CEC 2017 Comparative Experiments and Analysis

To evaluate the performance differences between EBOHO and other competing algorithms, the Mann–Whitney U test was used to analyze statistical significance. The results are represented by the symbols ‘+’, ‘≈’, and ‘−’, which indicate that EBOHO is significantly better than, is significantly different from, or is significantly worse than the specific competing algorithm, respectively. Additionally, the Friedman ranking test was used to assess the average rankings of each algorithm across different test sets, with the best-performing algorithm’s ranking displayed in bold.

**Figure 4:**
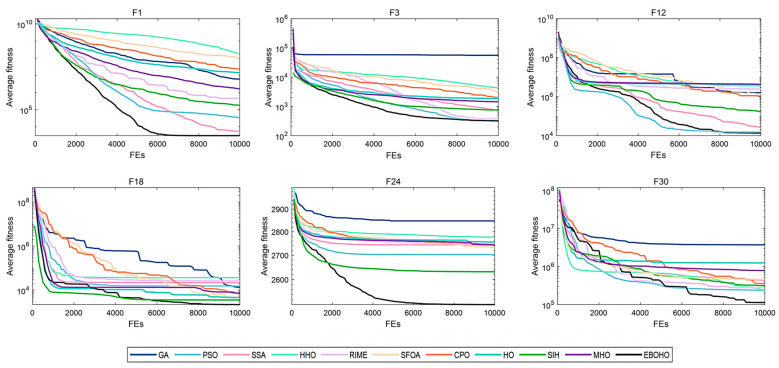
Convergence curves for 10-D CEC2017 benchmark functions, comparing the convergence speed and performance of EBOHO with other optimization algorithms on the 10D test problem.

**Figure 5:**
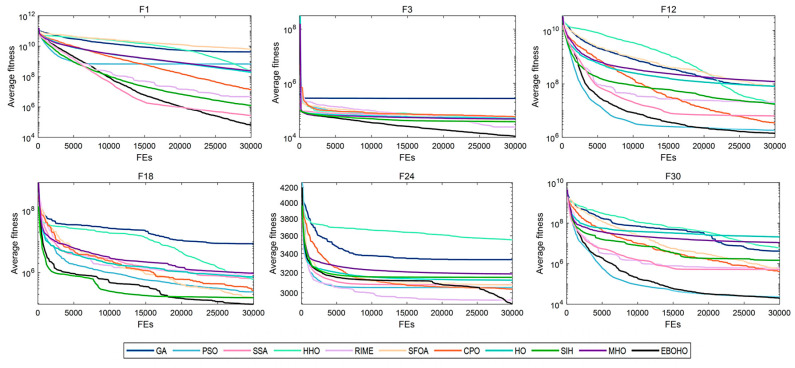
Convergence curves for 30-D CEC2017 benchmark functions, showing EBOHO’s performance on the 30D test problem and comparing it with other algorithms.

**Figure 6:**
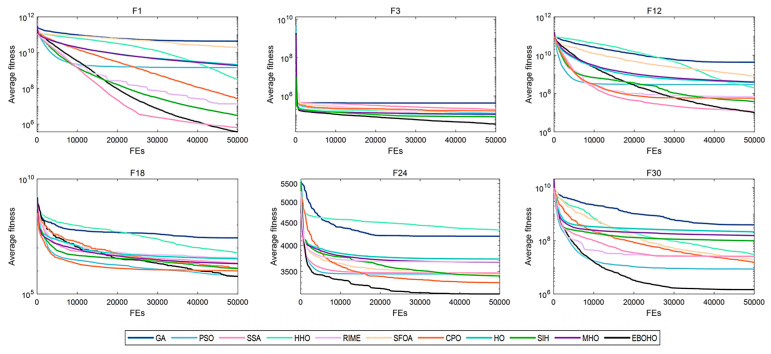
Convergence curves for 50-D CEC2017 benchmark functions, demonstrating EBOHO’s performance on the 50D test problem and highlighting its superiority in high-dimensional optimization.

**Figure 7:**
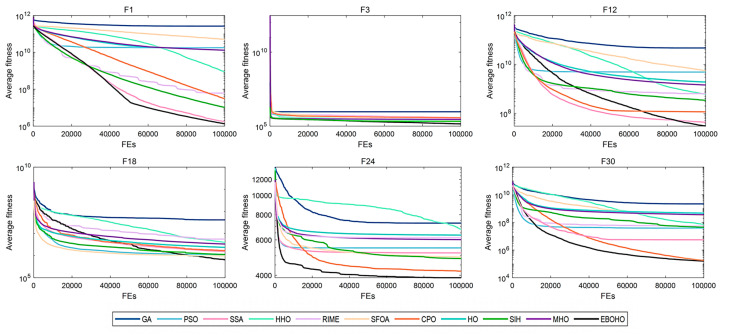
Convergence curves for 100-D CEC2017 benchmark functions, displaying the convergence behavior of EBOHO on the 100D test problem and validating its effectiveness in high-dimensional optimization.

Table 3 summarizes the statistical significance and average rankings of the algorithms in CEC 2017, with detailed results provided in Appendix B. Figure 4, Figure 5, Figure 6 and Figure 7 display the convergence curves of the optimizers for the representative functions in CEC 2017 (i.e., F1: unimodal function; F3: multimodal function; F12 and F18: hybrid functions; and F24 and F30: composite functions).

As shown in Table 3, EBOHO consistently outperforms other competitor algorithms across different dimensions in CEC 2017, demonstrating exceptional optimization capabilities. Furthermore, statistical analysis further validates the effectiveness and efficiency of EBOHO, emphasizing the importance of the unique strategies introduced in its algorithm design. The experimental results indicate that the synergistic effect of EBOHO’s three key strategies significantly enhances search performance. Additionally, EBOHO continues to improve solution quality in the later stages of optimization, highlighting its powerful exploitation ability, with particularly impressive performance in the optimization convergence curves.

More importantly, traditional high-citation optimization algorithms such as GA, PSO, SSA, HHO, RIME, and even other improved versions of HO, perform relatively weakly in comparison to EBOHO. This observation is consistent with the No Free Lunch (NFL) theorem, which states that no single algorithm can exhibit optimal performance across all types of problems. Nevertheless, EBOHO demonstrates outstanding performance across various standard benchmark tests, but it may still face certain challenges in specific problem applications, further emphasizing the necessity for customization and adaptation in future research for particular problems.

### 4.4. Ablation Experiment

To independently study the performance of the proposed strategies, we conducted ablation experiments on the CEC 2017 benchmark with 30D and 50D dimensions. We first define the abbreviations of the algorithms: HO: original standard HO algorithm, HO-Beta: HO + Beta function initialization, HO-EMO: HO + Elite-Mean Operator (EMO), HO-LIRL: HO + Lens Imaging Reverse Learning (LlRL), and EBOHO. Mann–Whitney U statistical tests were also performed. Table 4 summarizes the statistical significance and average rankings of the CEC 2017 benchmark test set under the ablation experiments, with detailed results provided in Appendix C.

The ablation experiments indicate that each strategy plays a specific role in enhancing the performance of the HO algorithm. However, when only a single strategy is introduced, the performance improvement is typically confined to a particular search phase—either benefiting the early exploration phase or promoting the later exploitation phase. This local optimization often results in the algorithm performing better in certain stages compared to others, leading to bottlenecks that limit the overall stable improvement in performance.

Thus, this phenomenon highlights the importance of a comprehensive algorithm design approach. A single strategy cannot simultaneously balance global diversity and local precision, while combining beta function initialization, the elite–mean operator (EMO), and Lens Imaging Reverse Learning (LlRL) enables the full utilization of each strategy’s strengths at different stages, forming a systematic collaborative search framework. This approach ensures a broad exploration in the initial stage and precise convergence optimization in the final stage, allowing EBOHO to maintain diversity in exploration while achieving accurate local development, thus achieving remarkable results on the CEC benchmark tests.

The performance degradation observed when any individual component is removed (see Table A8 and Table A9) confirms that each strategy contributes to the overall optimization process. Furthermore, the results show that the combination of all three strategies in EBOHO consistently outperforms their individual applications, demonstrating a synergistic effect that leads to robust and balanced optimization performance.

## 5. Numerical Experiments in Engineering Problems

This section studies the performance of the proposed EBOHO algorithm in real-world engineering design optimization problems. We use four classic engineering problems—the Pressure Vessel Design Problem (PVDP) [43], Tapered Column Design Problem (TCDP) [44], Truss Bridge Design Problem (TBTDP) [45], and Piston Rod Design Problem (PLDP) [46]—as the primary test cases. Numerical simulations are conducted for these problems, and the computational results are analyzed. Detailed descriptions of these problems are provided in Appendix E. Additionally, all the optimizers listed in Table 2 are used as competing algorithms, and each algorithm is independently run 30 times, with the stopping condition for each run being the maximum number of evaluations that reach 10,000.

### 5.1. Experiments in the Pressure Vessel Design Problem (PVDP)

**Figure 8:**
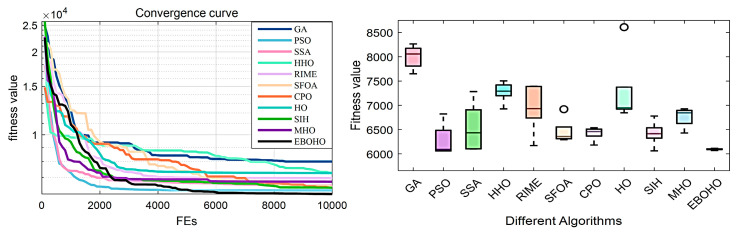
Convergence figures and boxplots for the Pressure Vessel Design Problem (PVDP), showing the optimization performance of EBOHO and demonstrating superior convergence compared to other algorithms.

PVDP: The objective of the Pressure Vessel Design Problem (PVDP) is to minimize the total cost while meeting production requirements. The four design variables include shell thickness, head thickness, internal radius, and vessel length (excluding the head). The specific model and equations are provided in Appendix D.1. The experimental results and statistical analysis are summarized in Table 5, with the convergence curves and box plots shown in Figure 8.

In the PVDP, the top three optimizers are EBOHO, PSO, and CPO. The proposed EBOHO outperforms the other algorithms across all indices, clearly demonstrating its superior performance compared to HO and its variants in solving the PVDP. This further confirms the strong potential of EBOHO for engineering applications.

### 5.2. Experiments in the Tubular Column Design Problem (TCDP)

**Figure 9:**
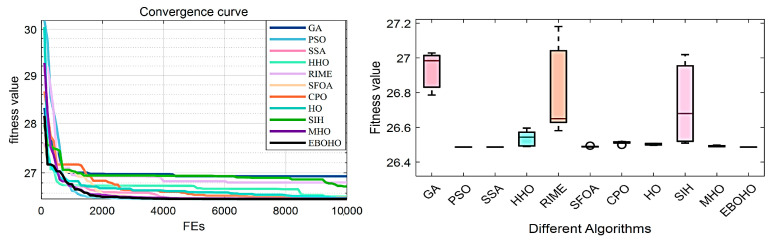
Convergence figures and boxplots for the Tapered Column Design Problem (TCDP), illustrating EBOHO’s performance on the Tapered Column Design Problem and emphasizing its precision and stability in optimization.

The Tapered Column Design Problem (TCDP) is an example of designing a tubular section column to support a compressive load at minimum cost. This example consists of two key variables: the average diameter of the column ($d=x_{1}$) and the thickness of the tube ($t=x_{2}$). The specific mathematical model and equations are provided in Appendix D.2. Table 6 summarizes the experimental results and statistical analysis of the optimizers in TCDP, while Figure 9 presents the convergence curves and box plots of each algorithm.

In this problem, the top three optimizers are EBOHO, MHO, and SFOA. Although the performance of these three optimizers is very close, the proposed EBOHO demonstrates a smaller standard deviation, suggesting slightly stronger consistency in multiple runs. While MHO and SFOA achieve comparable accuracy, EBOHO provides more stable results across independent trials.

### 5.3. Experiments in the Three-Bar Truss Design Problem (TBTDP)

**Figure 10:**
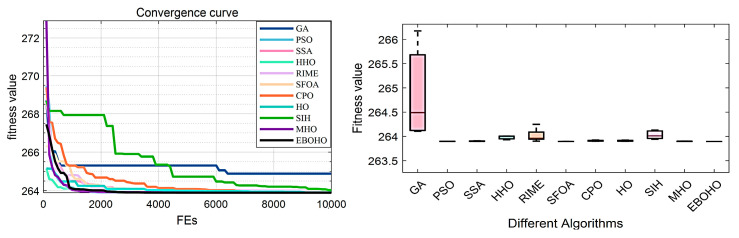
Convergence figures and boxplots for the Truss Bridge Design Problem (TBTDP), comparing EBOHO’s performance on the Truss Bridge Design Problem and showcasing its advantages over other algorithms.

In the Truss Bridge Design Problem (TBTDP), a common nonlinear fractional optimization problem in civil engineering, the objective is to minimize the volume of a three-bar truss while imposing three types of constraints on each truss component: buckling, deflection, and stress. The key parameters of this problem are $A_{1}$ and $A_{2}$, and the specific model and equations can be found in Appendix D.3. The experimental results and statistical analysis are summarized in Table 7, with the convergence curves and box plots presented in Figure 10.

In this problem, the top three optimizers are EBOHO, MHO, and SFOA. Although the performance of the top three optimizers is nearly identical in terms of accuracy, EBOHO maintains slightly better stability across experiments. This suggests that EBOHO is more robust under repeated trials, although MHO and SFOA remain competitive in this task.

### 5.4. Experiments in the Piston Lever Design Problem (PLDP)

**Figure 11:**
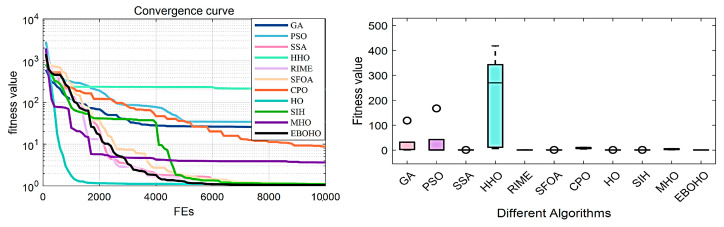
Convergence figures and boxplots for the Piston Rod Design Problem (PLDP), showing the optimization performance of EBOHO and comparing its convergence behavior and stability with other algorithms.

In the Piston Rod Design Problem (PLDP), the main objective is to minimize the oil volume when the piston rod rises to 45° according to the mathematical equations, in order to position the piston components $H(=x_{1})$, $B(=x_{2})$, $D(=x_{3})$, and $X(=x_{4})$. The specific model and equations can be found in Appendix D.4. The experimental results and statistical analysis are summarized in Table 8, with the convergence curves and box plots shown in Figure 11.

In solving the PLDP problem, the top three optimization algorithms are EBOHO, HO, and SSA. It is noteworthy that while the MHO algorithm performs excellently in the TCDP and TBTDP problems, its performance significantly declines in the PLDP problem. This result further validates the No Free Lunch Theorem, which states that no single optimization algorithm can perform optimally across all types of problems. The characteristics of different problems can lead to variations in the performance of different algorithms, and even the same algorithm may yield dramatically different results on different problems. Therefore, in practical applications, when selecting an optimization algorithm, adjustments and optimizations should be made based on the specific characteristics of the problem, rather than relying on the general applicability of one algorithm across all problems.

## 6. Application of EBOHO in Grounding Network Corrosion Diagnosis

Grounding network corrosion is a significant safety hazard in power systems, especially in harsh environments, where the corrosion of grounding networks is becoming increasingly severe [47]. As a critical component of power system protection, the corrosion of the grounding network not only increases grounding resistance, affecting system stability, but can also lead to grounding faults and equipment damage [48,49]. Currently, existing detection methods have many shortcomings, and traditional detection techniques often rely on manual large-scale excavation, which cannot achieve high-precision assessments and is susceptible to environmental factors and human errors. Therefore, researchers have attempted to establish corrosion diagnostic models based on electrical network methods, aiming to assess the degree of corrosion by analyzing changes in electrical parameters. However, this model involves nonlinear underdetermined systems of equations [50], making the solving process complex and potentially leading to insufficient diagnostic accuracy. Consequently, the development of a simple yet effective method to solve the grounding network corrosion diagnostic model has become crucial.

In this context, the proposed EBOHO algorithm provides an effective and easily implementable solution to address the challenges of grounding network corrosion diagnosis. In the following sections, Section 6.1 introduces the nonlinear underdetermined system model for grounding network corrosion diagnosis, and Section 6.2 presents the simulation and experimental results.

### 6.1. Development of the Corrosion Diagnosis System Model

**Figure 12:**
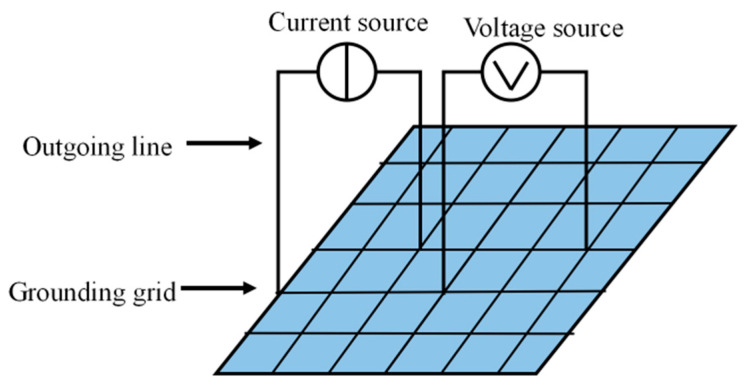
Demonstration illustrating the basic structure of a power system’s grounding network.

From an electrical perspective, the grounding network can be viewed as a purely resistive network composed of multiple grounding conductors and connection points [51], as shown in Figure 12. Its topological structure remains unchanged before and after corrosion; however, the values of branch resistances may change over time. Corrosion leads to an increase in the resistance of the conductor surfaces and connection points, which in turn affects the grounding performance [52].

For a grounding network with n branches and b branches, according to electrical network theory, we have the following equalities:

(28) $$G_{n}=AG_{b}A^{T}$$

(29) $$U_{n}=G_{n}^{-1}I_{n}$$

(30) $$U_{b}=A^{T}U_{n}$$

(31) $$I_{b}=G_{b}A^{T}U_{n}$$

where A is the adjacency matrix of the network, $G_{b}$ is the branch conductance matrix, $G_{n}$ is the node conductance matrix, $U_{n}$ is the node voltage matrix, $U_{b}$ is the branch voltage matrix, $I_{n}$ is the node current matrix, and $I_{b}$ is the branch current matrix. Additionally, according to Ohm’s law [53], the port impedance between network nodes i,j can be expressed as follows:

(32) $$R_{ij}=\frac{U_{\mathrm{ij}}}{I_{ij}}=f(R_{1},R_{2},\ldots R_{b})$$

The core of this method is to measure the port resistances between accessible nodes in the grounding network and then invert these measurements to obtain the actual values of the branch resistances. By comparing the differences between these actual resistance values and the nominal resistance values, it is possible to determine the degree of corrosion in the grounding network and the location where corrosion has occurred.

A DC current source is injected at the accessible node terminals of the grounding network. The topological structure of the grounding network remains unchanged before and after corrosion; the only change is that the branch resistance values affected by corrosion have varied. Therefore, using Thevenin’s theorem, it is known that:

(33) $$\Delta R_{ij}=\sum_{k=1}^{b}\Delta R_{k}I_{k}^{\prime }I_{k}/I_{0}^{2}$$

where $\Delta R_{ij}$ is the change in the port resistance at the accessible node terminals, $I_{k}^{\prime }$ is the branch current after corrosion, and $I_{k}$ is the branch current before corrosion. These three variables are known quantities and can be measured on-site, and $\Delta R_{k}$ represents the change in the branch resistance, which is the unknown quantity to be determined.

For a grounding network with m+1 accessible nodes (with one selected as the reference node), after injecting a DC current source, $I_{0}$, an m-dimensional corrosion diagnosis system of equations can be obtained as follows:

(34) $$\left\{\begin{array}{c} \Delta R_{ij(1)}=\sum_{k=1}^{b}\Delta R_{k}I_{k(1)}^{\prime }I_{k(1)}/I_{0}^{2}\\ \Delta R_{ij(2)}=\sum_{k=1}^{b}\Delta R_{k}I_{k(2)}^{\prime }I_{k(2)}/I_{0}^{2}\\ \vdots \\ \Delta R_{ij(m)}=\sum_{k=1}^{b}\Delta R_{k}I_{k(m)}^{\prime }I_{k(m)}/I_{0}^{2} \end{array}\right.$$

where $\Delta R_{ij(m)}$, $I_{0}$, $R_{k}$, and $I_{k}$ can be obtained through on-site testing and the design parameters of the substation grounding network. The corrosion branch current, $I_{k}^{\prime }$, contained in the port resistance of each accessible node is different, and the branch current, $I_{k}^{\prime }$, is determined by the post-corrosion branch resistance, $R_{k}^{\prime }$. Since there is a direct relationship between $I_{k}^{\prime }$ and $R_{k}^{\prime }$, they influence each other, resulting in the nonlinear nature of the system of equations.

The number of branches, b, in the grounding network is often greater than the number of nodes, n, while the number of accessible nodes, m, is always less than or equal to the total number of nodes, n, in the network. Therefore, it can be concluded that the number of equations, m, in the system is always less than the number of unknown branch resistances, b, meaning that the number of equations is fewer than the number of unknowns. As a result, this corrosion diagnosis system of equations is an underdetermined system.

Therefore, it is necessary to find an appropriate optimization algorithm to solve the underdetermined nonlinear corrosion diagnosis system of equations in order to obtain more accurate and realistic solutions for branch resistances.

### 6.2. Model Simulation Experiments

This paper will use MATLAB to build a grounding network simulation model, where the corresponding resistance values for the corroded branches will be set. Subsequently, the proposed EBOHO algorithm will be applied to solve the model. The corrosion degree will be evaluated by comparing the final results (detailed in Table 9), thereby verifying the reliability of the algorithm in grounding network corrosion diagnosis.

#### 6.2.1. Single-Branch Experimental Test

**Figure 13:**
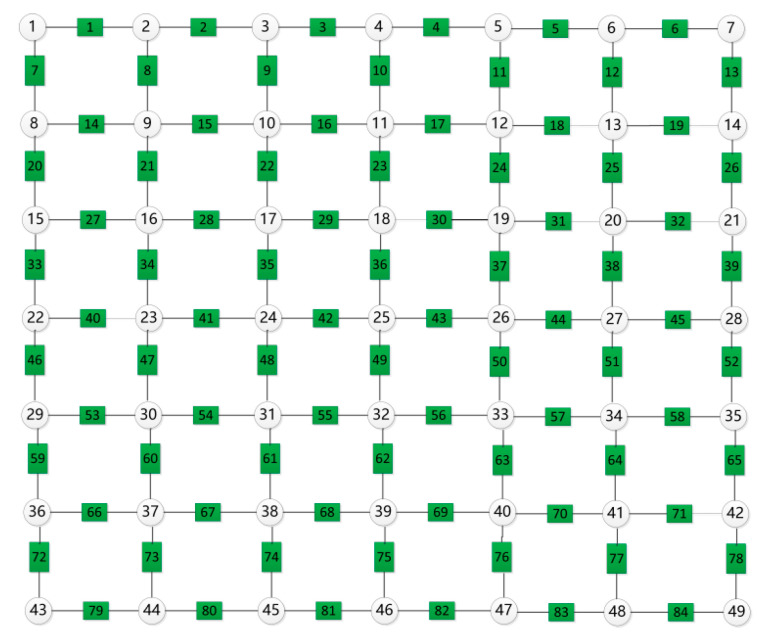
Diagram of a 7 × 7 grounding network showing the configuration of nodes and branches.

The simulation builds a 7 × 7 grounding network, including 49 nodes and 84 branches, and the nodes and branches are numbered according to the principle of left-to-right and top-to-bottom, as shown in Figure 13.

The initial resistance of all the branch circuits is set to 1 mΩ, and the resistance of branch circuit No. 42 is adjusted to 10 mΩ, 20 mΩ, and 300 mΩ in order to simulate the three scenarios of moderate corrosion, severe corrosion, and disconnection, respectively. The deviation of the algorithm diagnosis results from the real value is expressed as relative error.

In a 7 × 7 grounding network, there are 84 branches, i.e., the problem dimension is 84 dimensions. The parameters of the EBOHO algorithm are as follows:

- Population size: 100;
- Maximum number of function evaluations: 10,000.

Each group of tests was run independently 30 times, and the optimal solution was taken as the corrosion diagnosis value for the current time in order to reduce the influence of randomness of the intelligent algorithm and to obtain more reliable performance indices. The abovementioned EBOHO parameter settings were used for all subsequent grounding network experiments.

**Figure 14:**
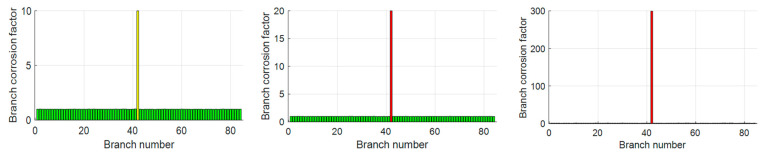
Single-branch diagnostic result displaying the diagnostic accuracy of EBOHO for a single branch in the grounding network.

The experimental results show that when the resistance of branch 42 is set to 10 mΩ, 20 mΩ, and 300 mΩ, the diagnostic values given by the algorithm are 9.9882 mΩ, 19.9957 mΩ, and 299.1452 mΩ, respectively, the three conditions corresponding to the determination of moderate corrosion, heavy corrosion, and disconnection are accurate, and the relative error is much lower than the upper limit of the engineering tolerance, which proves that the algorithm is capable of solving the single-branch corrosion diagnosis problem reliably and with high accuracy. The experimental results are shown in Figure 14.

#### 6.2.2. Multi-Branch Experimental Test

**Figure 15:**
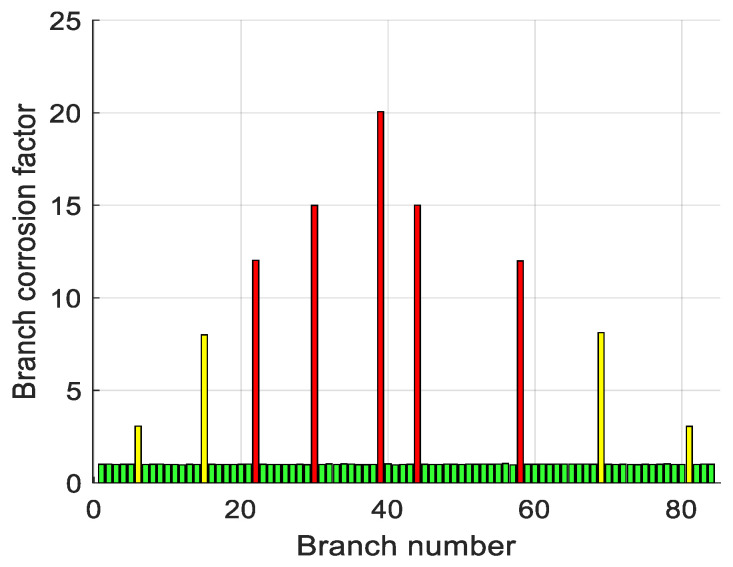
Multi-channel diagnostic results, presenting the performance of EBOHO in diagnosing multiple corroded branches simultaneously.

In view of the common situation of the simultaneous corrosion of multiple branches at engineering sites, this section still takes the 7 × 7 grounding network as the object to carry out numerical simulation to evaluate the diagnostic performance of the proposed method in multi-branch corrosion scenarios, with the simulation parameters described below; each corroded branch and its post-corrosion resistance value are shown in Table 10. The simulation parameters are set as follows: the grounding resistance of the normal branch is uniformly 1 mΩ; the corroded branches and their resistance values after corrosion are shown in Table 10; and the diagnostic results based on the above parameters for the fault injection and inversion tests are shown in Figure 15, which can reflect the corrosion degree of the different branches and the localization accuracy.

Engineering practice generally accepts the relative error of grounding network corrosion diagnosis as ≤5%. The abovementioned simulation and experimental results of the error satisfy or exceed the threshold, fully verify the accuracy and reliability of the proposed algorithm, and show that it can be applied to the field engineering and other broad prospects.

#### 6.2.3. Algorithm Comparison and Performance Evaluation Analysis

In order to further assess the strong application prospects of EBOHO for grounding network corrosion diagnosis applications, an algorithm performance comparison can be carried out, and we select the classical intelligent algorithm, PSO, the highly referenced algorithm, GWO, the latest algorithm, SFOA, the original algorithm, HO, and the variant algorithms, SIH and MHO, for comparison.

**Figure 16:**
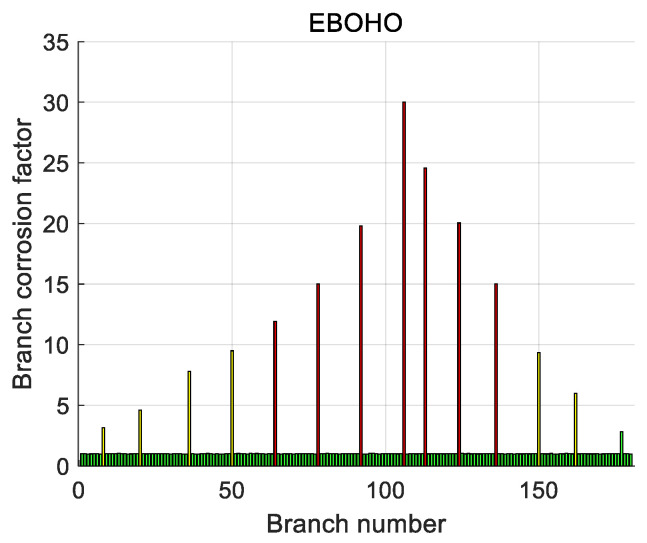
EBOHO diagnostic result chart visualizing EBOHO’s ability to detect corrosion locations and estimate resistance values in a large-scale grounding network.

We call the branch that originally has no corrosion. Still, the error of the diagnosis result is more than 3 mΩ as a misdiagnosis branch, and we use the high-dimensional 10 × 10 grounding network for comparison, with a total of 100 nodes and 180 branches. Addtionally, the corrosion of the grounding network is set as in Table 11, and the resistance of the rest of the branches is still 1 mΩ. The diagnosis results of each algorithm are shown in Table 11. We can see that EBOHO still has obvious superiority in the 10 × 10 grounding network diagnosis problem. The overall diagnosis effect of SFOA, HO, SIH, and MHO is general, and these algorithms result in a large number of misdiagnoses. It can be seen that these algorithms are not suitable for the high-dimensional grounding network corrosion diagnosis problem, which also proves that the NFL theorem is correct again. The corrosion branch solution of EBOHO is shown in Figure 16, and the comparative algorithms’ solution diagrams are shown in Appendix E. The diagnosis effect of PSO and GWO is also acceptable, and the results of these algorithms are shown in Table 11. The diagnostic effect of GWO is acceptable, and there is no misdiagnosis. However, compared with our proposed EBOHO algorithm, there is still a gap, and some appropriate improvements can be made to make it more suitable for underdetermined problem solving.

Combined with the experimental results, our proposed EBOHO algorithm exhibits great competitiveness in both low-dimensional diagnostic problem solving for grounding networks and high-dimensional problems, which shows that the performance of this algorithm is stable and superior. It not only significantly outperforms traditional methods and representative intelligent algorithms in terms of solution speed but also shows excellent advantages in convergence accuracy and robustness for complex problems, which fully verifies its potential and applicability in practical applications. This indicates that EBOHO is well adapted to problems of varying scales and complexities, providing an efficient and reliable solution for grounding network corrosion diagnosis. While the 10 × 10 grid simulations demonstrate promising performance, the scalability of EBOHO to larger network sizes (such as 100 × 100 or larger) remains an important consideration for real-world applications. The current results show that EBOHO performs well under the given conditions; however, further research is needed to optimize its computational efficiency and explore its robustness when applied to larger, more complex grid systems. Future work will focus on investigating parallelization techniques and adaptive parameter control mechanisms to ensure that EBOHO can scale effectively for large-scale applications in practical scenarios.

## 7. Conclusions

In this paper, we propose a multi-strategy improved hippo optimization algorithm, EBOHO, which combines beta-distributed population initialization, the elite–mean operator, and lens imaging inverse learning for the underdetermined nonlinear optimization problem of the corrosion diagnosis of grounding networks. The three mechanisms synergistically improve the global search diversity and late convergence speed while maintaining a time complexity comparable to that of the mainstream meta-heuristics, which lays the foundation for the robustness and efficiency of the algorithm in large-scale complex optimization problems.

For numerical validation, EBOHO achieves the best average ranking in the CEC2017 multidimensional benchmarking test and shows a significant advantage over most of the competing algorithms according to the Mann–Whitney U test. Further, for solving four classical engineering design models, including the pressure vessel, pipe column, three-bar truss, and piston rod models, EBOHO is ranked first with the lowest objective value.

For practical applications, this paper embeds EBOHO into the grounding network corrosion diagnosis model established by the electrical network method. In the 7 × 7 and 10 × 10 high-dimensional grounding network simulation, the algorithm controls the relative error within 2%, which is much better than the generally acceptable threshold of ≤5%. The comparative algorithms generally suffer from misdiagnosis or accuracy degradation in high-dimensional scenarios. The abovementioned results fully demonstrate that EBOHO can realize the accurate inversion of corrosion location and extent with minimal excavation and can be directly extended to online monitoring in the field.

Therefore, EBOHO not only provides a new intelligent solution paradigm for underdetermined optimization theoretically but also verifies its excellent performance and engineering value through multi-level experiments, which provides strong technical support for the rapid assessment of the corrosion of power system grounding networks and other large-scale network parameter inversion problems. Beyond corrosion diagnosis, EBOHO also shows promise for extension to other diagnostic or reliability assessment tasks in power systems, such as fault impedance estimation or transformer condition monitoring.

Nevertheless, like most metaheuristic algorithms, EBOHO has some limitations. Its performance can be sensitive to parameter settings, such as population size and iteration number, and further validation is needed to assess its effectiveness in ultra-high-dimensional or real-time optimization problems. To overcome these challenges, future work will focus on developing adaptive parameter control mechanisms to dynamically adjust these settings based on problem characteristics. Additionally, we plan to explore hybridizing EBOHO with machine learning models to enhance its ability to handle complex, nonlinear relationships in real-world data. Finally, the deployment of parallel or distributed versions of EBOHO will be investigated, enabling large-scale, high-speed optimization for industrial applications and ensuring scalability in more challenging optimization problems.

## Figures and Tables

**Table 1:** Computational complexity of typical MAs.

MAs	Complexity
GA	O(T×N×D)
PSO	O(T×N×D)
SSA	O(T×N×D)
RIME	O(T×N×D)
HO	O(T×N×D)
SIH	O(T×N×D)
MHO	O(T×N×D)
EBOHO	O(T×N×D)

**Table 2:** Parameter settings of the comparison algorithms.

MAs	Parameters	Value
GA	Probability of crossover $p_{c}$ Probability of mutation $p_{m}$	0.6 0.05
PSO	Inertia factor ω Acceleration coefficients $c_{1}$ and $c_{2}$ Max. and min. speed	1 2.05 2, −2
SSA	P_percent	0.2
HHO	No Parameters	
RIME	Parameter ω	5
SFOA	Parameter GP	0.5
CPO	Convergence rate alpha Trade-off ratio	0.2 0.8
HO	No Parameters	
SIH	No Parameters	
MHO	Chaos control parameter	1
EBOHO	Beta distribution parameter a=b	0.8

**Table 3:** Summarized experimental results of comparison experiments in CEC2017.

MAs	10Dim		30Dim		50Dim		100Dim	
	+/≈/−	Avg. Ranks	+/≈/−	Avg. Ranks	+/≈/−	Avg. Ranks	+/≈/−	Avg. Ranks
GA	29/0/0	9.65	29/0/0	10.00	29/0/0	10.38	29/0/0	10.79
PSO	29/0/0	5.93	29/0/0	4.66	29/0/0	5.45	28/0/1	6.52
SSA	28/1/0	6.52	27/1/1	5.31	26/1/2	5.35	28/0/1	4.72
HHO	29/0/0	9.21	28/1/0	8.48	29/0/0	7.72	29/0/0	7.31
RIME	25/1/3	5.66	24/0/5	3.86	22/1/6	4.52	24/0/5	4.21
SFOA	27/0/2	6.17	27/0/2	7.76	29/0/0	7.79	28/0/1	7.69
CPO	25/0/4	4.9	28/0/1	5.03	27/0/2	4.38	27/0/2	4.62
HO	29/0/0	7.1	29/0/0	7.52	29/0/0	7.34	29/0/0	7.24
SIH	22/3/4	3.28	27/1/1	4.21	25/1/3	4.17	25/0/4	3.9
MHO	28/0/1	6.03	29/0/0	7.83	28/1/0	7.38	29/0/0	7.21
EBOHO	~	1.55	~	1.34	~	1.52	~	1.79

**Table 4:** Summary of the ablation experiment results of CEC2017 at 30D and 50D.

MAs	30Dim		50Dim	
	+/≈/−	Avg. Ranks	+/≈/−	Avg. Ranks
HO	~	4.45	~	4.62
HO-Beta	4/3/22	3.69	5/1/23	3.75
HO-EMO	2/2/25	2.20	1/0/28	1.97
HO-LIRL	6/3/20	3.52	4/1/24	3.45
EBOHO	0/0/29	1.14	0/1/28	1.21

**Table 5:** PVDP experimental results and statistical analysis.

MAs	Mean	Std	Best	Worst
GA	7.9955E+03+	2.4222E+02	7.6497E+03	8.2638E+03
PSO	6.2802E+03+	3.2912E+02	6.0597E+03	6.8204E+03
SSA	6.5399E+03+	5.0084E+02	6.0994E+03	7.2818E+03
HHO	7.2788E+03+	2.1674E+02	6.9247E+03	7.5019E+03
RIME	6.9610E+03+	4.9854E+02	6.1700E+03	7.3920E+03
SFOA	6.4634E+03+	2.6021E+02	6.2921E+03	6.9193E+03
CPO	6.4206E+03+	1.4017E+02	6.1804E+03	6.5358E+03
HO	7.2631E+03+	7.5407E+02	6.8463E+03	8.6094E+03
SIH	6.4240E+03+	2.5380E+02	6.0624E+03	6.7787E+03
MHO	6.7543E+03+	2.0186E+02	6.4296E+03	6.9245E+03
EBOHO	**6.0936E+03**	1.6165E+01	6.0675E+03	6.1120E+03

Bold values indicate the optimal values, which are consistently used throughout the manuscript.

**Table 6:** TCDP experimental results and statistical analysis.

MAs	Mean	Std	Best	Worst
GA	2.6876E+01+	2.5860E−01	2.6646E+01	2.7278E+01
PSO	2.6492E+01+	3.2140E−05	2.6492E+01	2.6492E+01
SSA	2.6491E+01+	2.0000E−04	2.6491E+01	2.6491E+01
HHO	2.6612E+01+	1.3560E−01	2.6501E+01	2.6795E+01
RIME	2.6676E+01+	2.3840E−01	2.6497E+01	2.7062E+01
SFOA	2.6488E+01≈	1.9000E−03	2.6487E+01	2.6491E+01
CPO	2.6498E+01+	7.2000E−03	2.6492E+01	2.6510E+01
HO	2.6499E+01+	1.1600E−02	2.6487E+01	2.6513E+01
SIH	2.6634E+01+	1.0910E−01	2.6532E+01	2.6804E+01
MHO	2.6487E+01≈	2.4000E−03	2.6486E+01	2.6492E+01
EBOHO	**2.6486E+01**	2.2240E−07	2.6486E+01	2.6486E+01

Bold values indicate the optimal values, which are consistently used throughout the manuscript.

**Table 7:** TBTDP experimental results and statistical analysis.

MAs	Mean	Std	Best	Worst
GA	2.6488E+02+	9.2160E−01	2.6410E+02	2.6617E+02
PSO	2.6394E+02+	1.9000E−03	2.6390E+02	2.6396E+02
SSA	2.6397E+02+	6.2000E−03	2.6390E+02	2.6399E+02
HHO	2.6398E+02+	3.4400E−02	2.6393E+02	2.6401E+02
RIME	2.6402E+02+	1.3590E−01	2.6390E+02	2.6425E+02
SFOA	2.6390E+02≈	7.0000E−04	2.6390E+02	2.6390E+02
CPO	2.6391E+02≈	1.0200E−02	2.6390E+02	2.6393E+02
HO	2.6391E+02≈	1.0400E−02	2.6390E+02	2.6392E+02
SIH	2.6403E+02+	8.4100E−02	2.6394E+02	2.6413E+02
MHO	2.6390E+02≈	2.6000E−03	2.6390E+02	2.6390E+02
EBOHO	**2.6390E+02**	2.2234E−07	2.6390E+02	2.6390E+02

Bold values indicate the optimal values, which are consistently used throughout the manuscript.

**Table 8:** PLDP experimental results and statistical analysis.

MAs	Mean	Std	Best	Worst
GA	2.5817E+01+	5.1948E+01	1.3026E+00	1.1873E+02
PSO	3.4341E+01+	7.4423E+01	1.0574E+00	1.6747E+02
SSA	1.0901E+00+	5.2700E−02	1.0578E+00	1.1835E+00
HHO	2.0520E+02+	1.8595E+02	6.5411E+00	4.1788E+02
RIME	1.1487E+00+	1.0210E−01	1.0639E+00	1.2900E+00
SFOA	1.1105E+00+	5.0600E−02	1.0803E+00	1.2002E+00
CPO	8.6997E+00+	2.6476E+00	4.7468E+00	1.1096E+01
HO	1.0653E+00+	5.3000E−03	1.0625E+00	1.0747E+00
SIH	1.1039E+00+	7.9200E−02	1.0656E+00	1.2455E+00
MHO	3.6600E+00+	1.8690E+00	1.6947E+00	5.6477E+00
EBOHO	**1.0577E+00**	2.0000E−04	1.0575E+00	1.0580E+00

Bold values indicate the optimal values, which are consistently used throughout the manuscript.

**Table 9:** Corrosion visualization display standard.

Degree of Corrosion	Branch Change Multiple	Color of the Branch Resistor
Mild corrosion	1~3	green
Moderate corrosion	3~10	yellow
Severe corrosion (including fracture)	>10	red

**Table 10:** Multi-branch resistance corrosion data.

Branch Line Number	Resistance After Corrosion	Diagnostic Resistance	Relative Error (%)
6	3.0	3.0576	1.920
15	8.0	7.9982	0.023
22	12.0	12.0285	0.238
30	15.0	14.9981	0.013
39	20.0	20.0572	0.286
44	15.0	15.0026	0.017
58	12.0	11.9901	0.083
69	8.0	8.1190	1.488
81	3.0	3.0509	1.697

**Table 11:** Corrosion data results of 10 × 10 branch lines.

Branch Number	True Resistance	Diagnostic Resistance						
		PSO	GWO	SFOA	HO	SIH	MHO	EBOHO
8	3	3.4025	3.1323	2.9231	2.9646	2.9586	2.7641	3.1372
20	5	4.7645	4.7848	3.7479	3.8852	4.6728	3.2693	4.8917
36	8	8.2118	8.2351	3.5426	7.2529	6.1815	7.3862	7.8858
50	10	10.2953	9.7710	6.4889	8.9560	9.8553	7.2180	9.5924
64	12	12.2135	11.9034	10.4510	5.4854	9.8505	5.5589	11.9281
78	15	15.1399	14.7941	7.7070	13.0104	7.9277	14.3130	15.0156
92	20	19.9439	20.5965	9.1061	20.3269	15.9742	15.2076	19.7953
106	30	30.2026	29.9857	31.0000	30.6854	29.9786	30.3863	30.0201
113	25	25.2120	25.0538	9.5446	25.9098	23.0524	21.3341	24.5832
124	20	19.8075	20.0420	7.7759	19.2009	12.7360	11.9914	20.0429
136	15	14.8548	14.3886	7.0399	12.2828	12.1704	16.4047	15.0088
150	10	9.4955	9.9709	5.9616	6.5244	9.3958	7.4662	9.5356
162	6	6.0948	5.8683	4.6379	4.1905	4.5949	4.4368	5.9706
177	3	3.4827	3.1366	2.9823	3.0886	3.0928	3.2005	2.8284

**Figure A1:**
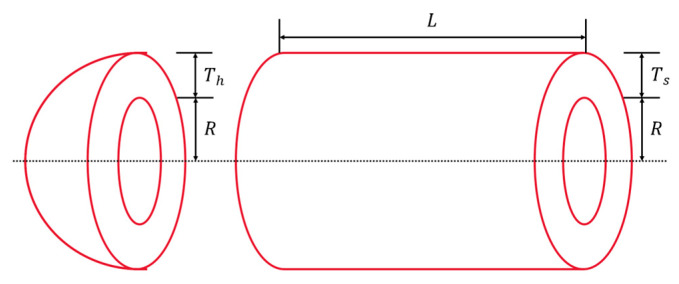
Demonstration of the Pressure Vessel Design Problem (PVDP).

**Figure A2:**
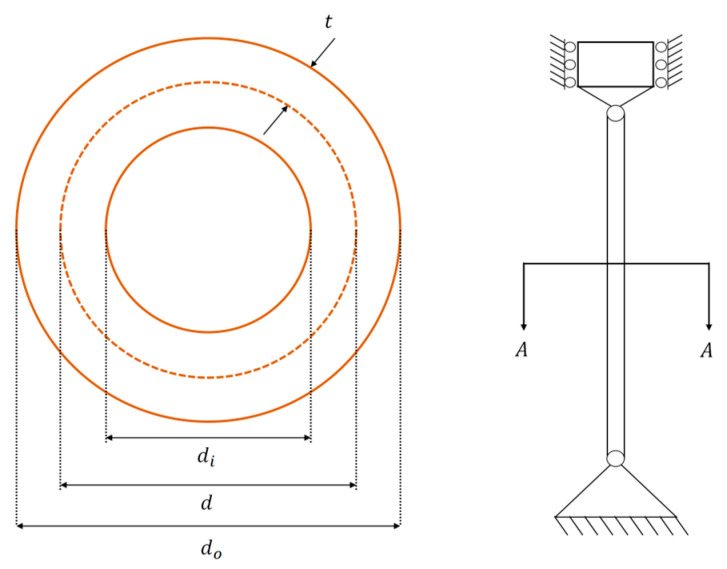
Demonstration of the Tubular Column Design Problem (TCDP).

**Figure A3:**
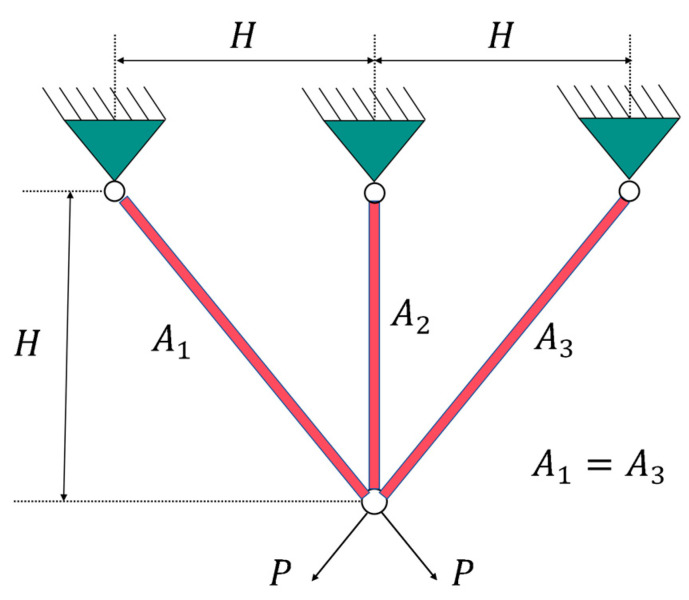
Demonstration of the Three-Bar Truss Design Problem (TBTDP).

**Figure A4:**
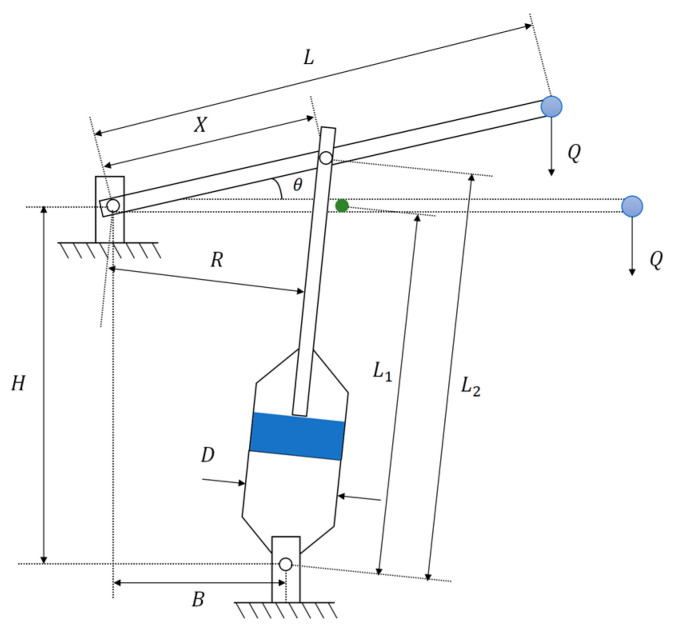
Demonstration of the Piston Lever Design Problem (PLDP).

**Figure A5:**
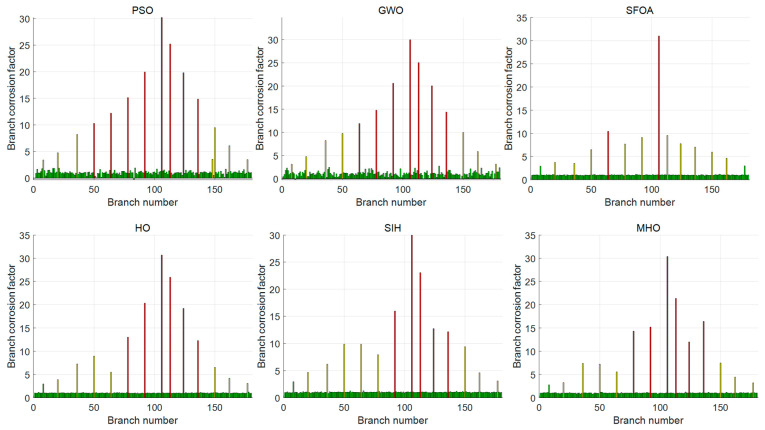
Results of the 10 × 10 comparison experiment for the remaining algorithms.

## Data Availability

Data are contained within the article.

## Appendix A. Basic Information on the CEC Benchmarks

Table A1 summarizes basic information on the CEC2017 benchmarks.

**Table A1:** CEC2017 benchmark: Uni. = unimodal function, Multi. = simple multimodal function, Hybrid. = hybrid function, Comp. = composition function.

No.	Func.	Feature.	Optimum.
f1	Shifted and Rotated Bent Cigar function	Uni.	100
f3	Shifted and Rotated Rastrigin function	Multi.	300
f4	Shifted and Rotated Expanded Scaffer’s F6 function		400
f5	Shifted and Rotated Lunacek Bi Rastrigin function		500
f6	Shifted and Rotated Lunacek BiRastrigin function		600
f7	Shifted and Rotated Non-Continuous Rastrigin function		700
f8	Shifted and Rotated Levy function		800
f9	Shifted and Rotated Schwefel function		900
f10	Hybrid function 1 (N = 3)	Hybrid.	1000
f11	Hybrid function 2 (N = 3)		1100
f12	Hybrid function 3 (N = 3)		1200
f13	Hybrid function 4 (N = 4)		1300
f14	Hybrid function 5 (N = 4)		1400
f15	Hybrid function 6 (N = 4)		1500
f16	Hybrid function 6 (N = 5)		1600
f17	Hybrid function 6 (N = 5)		1700
f18	Hybrid function 6 (N = 5)		1800
f19	Hybrid function (N = 6)		1900
f20	Composition function 1 (N = 3)	Comp.	200
f21	Composition function 2 (N = 3)		2100
f22	Composition function 3 (N = 4)		2200
f23	Composition function 4 (N = 4)		2300
f24	Composition function 5 (N = 5)		2400
f25	Composition function 6 (N = 5)		2500
f26	Composition function 7 (N = 6)		2600
f27	Composition function 8 (N = 6)		2700
f28	Composition function 9 (N = 3)		2800
f29	Composition function 10 (N = 3		2900
f30	Composition function 11 (N = 3)		3000
Search range: [−100,100]^D^			

## Appendix B. Results of Comparison Experiments on CEC2017

Table A2, Table A3, Table A4 and Table A5 summarize the detailed results of optimizers on CEC2017. Table A6 and Table A7 summarize the running times of the 30D and 50D tests of CEC2017.

**Table A2:** Detailed results of optimizers on 10-D CEC2017.

		GA	PSO	SSA	HHO	RIME	SOFA	CPO	HO	SIH	MHO	EBOHO
F1	std	4.6429E+06	4.1751E+03	6.5192E+03	1.2970E+08	5.1490E+05	4.0395E+07	1.2186E+07	3.6030E+07	1.5714E+05	1.0355E+06	3.9711E+04
	mean	6.0670E+06+	3.3385E+04+	5.4227E+03+	1.6729E+08+	4.5774E+05+	1.0869E+08+	2.2826E+07+	1.4179E+07+	1.8331E+05+	1.6180E+06+	**3.0534E+03**
F3	std	3.0389E+04	2.3147E+01	4.4173E+02	8.5699E+02	5.2195E+01	1.4994E+03	6.9404E+02	9.7638E+02	1.7111E+01	9.4774E+02	4.1752E+02
	mean	5.6076E+04+	3.2585E+02+	7.2952E+02+	4.3192E+03+	3.8190E+02+	3.1318E+03+	2.0253E+03+	1.8170E+03+	7.4826E+02+	1.4353E+03+	**3.2148E+02**
F4	std	4.5817E+01	1.7125E+00	2.6204E+01	5.7847E+00	1.2853E+00	7.7746E+00	1.5122E+00	3.1918E+01	2.2047E+00	3.5455E+01	2.2756E+01
	mean	4.7942E+02+	4.0673E+02+	4.1892E+02+	4.1015E+02+	4.0615E+02+	4.1653E+02+	4.0950E+02+	4.2926E+02+	4.0546E+02+	4.2055E+02+	**4.0395E+02**
F5	std	8.7624E+00	1.1576E+01	1.3272E+01	1.0589E+01	7.7168E+00	6.5658E+00	7.3681E+00	1.2121E+01	1.1996E+01	1.2665E+01	1.8051E+01
	mean	5.6449E+02+	5.1881E+02+	5.3406E+02+	5.6992E+02+	5.4536E+02+	5.4950E+02+	5.3444E+02+	5.3039E+02+	5.3830E+02+	5.3532E+02+	**5.1694E+02**
F6	std	1.0353E+01	9.1686E-01	6.9958E+00	3.7642E+00	5.4086E-01	3.7160E+00	1.1455E+00	9.8644E+00	9.0225E+00	7.6477E+00	8.2703E+00
	mean	6.4738E+02+	6.1892E+02+	6.3752E+02+	6.3470E+02+	6.2191E+02+	6.1407E+02+	6.1554E+02+	6.1749E+02+	6.2953E+02+	6.1726E+02+	**6.1024E+02**
F7	std	1.7088E+01	8.8359E+00	2.3793E+01	1.8341E+01	7.3461E+00	1.0902E+01	7.1891E+00	1.9343E+01	1.0509E+01	1.6444E+01	1.6583E+01
	mean	7.8967E+02+	7.8322E+02+	7.7158E+02+	7.9661E+02+	7.5227E+02+	7.8540E+02+	7.4924E+02+	7.6184E+02+	7.4176E+02+	7.5595E+02+	**7.3218E+02**
F8	std	1.3954E+01	8.5331E+00	9.9113E+00	6.3496E+00	1.0716E+01	4.9524E+00	4.0833E+00	6.9607E+00	9.0758E+00	4.8309E+00	5.2956E+00
	mean	8.6261E+02+	8.3868E+02+	8.2339E+02+	8.3440E+02+	8.2128E+02+	8.5632E+02+	8.3226E+02+	8.3874E+02+	8.2141E+02+	8.2005E+02+	**8.1710E+02**
F9	std	1.8056E+01	1.4326E-01	1.4742E+02	8.9265E+01	2.4050E+00	1.8995E+02	8.5353E+00	1.2271E+02	2.5457E+01	2.6988E+02	9.6172E+01
	mean	9.3161E+02+	9.2305E+02+	1.0440E+03+	1.3904E+03+	**9.0225E+02-**	1.1772E+03+	9.2167E+02+	1.0706E+03+	9.1771E+02+	1.1954E+03+	9.0655E+02
F10	std	2.3325E+02	3.6616E+02	3.1931E+02	3.1806E+02	2.3855E+02	3.3798E+02	2.0188E+02	2.5893E+02	1.4165E+02	1.4055E+02	2.7811E+02
	mean	2.4676E+03+	1.9299E+03+	1.9817E+03+	2.2895E+03+	1.8751E+03+	2.2439E+03+	2.3738E+03+	1.8513E+03+	**1.6863E+03-**	1.8867E+03+	1.7865E+03
F11	std	4.0869E+03	2.3550E+01	2.3659E+01	2.1434E+01	6.8985E+00	1.8838E+01	8.6611E+00	7.5300E+01	2.6344E+01	4.7849E+01	2.1908E+01
	mean	3.5859E+03+	1.1881E+03+	1.2336E+03+	1.1501E+03+	1.4115E+03+	1.1592E+03+	1.1774E+03+	1.1952E+03+	1.1487E+03+	1.1989E+03+	**1.1466E+03**
F12	std	1.9960E+06	6.5927E+03	1.8811E+04	2.5821E+06	3.6797E+06	7.2252E+05	4.7280E+05	3.7072E+06	1.8321E+05	4.8905E+06	1.1891E+04
	mean	1.6027E+06+	1.8558E+04+	2.6956E+04+	3.5179E+06+	2.4831E+06+	1.3654E+06+	1.0991E+06+	4.1744E+06+	1.7177E+05+	4.2573E+06+	**1.5396E+04**
F13	std	8.3391E+03	6.5263E+03	2.7371E+03	1.0434E+03	6.9014E+03	4.7527E+02	1.2467E+03	3.7282E+03	1.8059E+03	5.6593E+03	3.6441E+03
	mean	1.1658E+04+	8.1387E+03+	4.5345E+03+	4.1497E+04+	2.2554E+04+	2.2597E+03+	4.1271E+03+	7.9238E+03+	3.5298E+03+	8.2858E+03+	**2.1395E+03**
F14	std	3.3226E+03	2.9603E+03	2.6320E+03	1.3749E+03	9.2348E+02	1.2145E+01	6.1818E+00	2.4278E+01	1.7572E+01	2.2771E+01	2.4706E+01
	mean	3.4437E+03+	3.5289E+03+	4.1064E+03+	2.3204E+03+	2.1447E+03+	1.4624E+03+	1.4567E+03≈	1.5000E+03+	1.4973E+03+	1.4903E+03+	**1.4557E+03**
F15	std	8.9271E+03	2.9655E+03	5.8628E+03	1.8048E+03	5.1806E+03	4.1848E+01	2.7192E+01	8.9960E+02	3.4282E+02	1.5081E+03	1.1404E+03
	mean	1.4449E+04+	5.2610E+03+	6.5896E+03+	6.5903E+03+	5.8538E+03+	1.6524E+03-	**1.5828E+03-**	2.8775E+03+	2.1406E+03+	3.6698E+03+	1.7218E+03
F16	std	1.1081E+02	1.2629E+02	1.1018E+02	1.1809E+02	9.7818E+01	6.1430E+01	6.8340E+01	1.4153E+02	7.3813E+01	1.0552E+02	1.0817E+02
	mean	1.8239E+03+	1.7861E+03+	1.7238E+03+	1.9969E+03+	1.7602E+03+	1.7199E+03+	1.7162E+03-	1.8033E+03+	**1.7117E+03-**	1.7483E+03+	1.7185E+03
F17	std	2.2463E+01	5.2917E+01	4.8690E+01	1.0101E+01	1.9614E+01	2.4423E+01	9.2391E+00	1.8190E+01	9.6730E+00	1.5458E+01	1.7981E+01
	mean	1.7677E+03+	1.7824E+03+	1.7715E+03+	1.8335E+03+	**1.7457E+03-**	1.7981E+03+	1.7567E+03+	1.7670E+03+	1.7587E+03+	1.7644E+03+	1.7481E+03
F18	std	8.9992E+03	1.1413E+04	1.6364E+04	2.6048E+03	1.3356E+04	1.4241E+03	4.0353E+03	2.4126E+03	3.5067E+02	3.9815E+03	1.5470E+03
	mean	1.3479E+04+	1.5323E+04+	2.1961E+04+	3.6847E+04+	2.6373E+04+	4.2445E+03+	7.1093E+03+	4.6432E+03+	**2.2014E+03-**	7.2820E+03+	3.5745E+03
F19	std	6.9545E+03	7.8276E+03	3.4998E+03	5.7119E+02	3.3528E+03	4.0500E+01	2.8387E+01	2.9400E+03	6.0910E+02	2.4904E+03	3.7130E+03
	mean	1.0419E+04+	1.0032E+04+	4.6227E+03+	1.7241E+04+	1.0918E+04+	1.9951E+03-	**1.9654E+03-**	4.7368E+03+	2.4668E+03+	3.6553E+03+	2.0450E+03
F20	std	4.2328E+01	5.1819E+01	8.1260E+01	4.7253E+01	1.0524E+01	2.1907E+01	1.3712E+01	2.4532E+01	3.7420E+01	3.5131E+01	6.0125E+01
	mean	2.1405E+03+	2.0742E+03+	2.1304E+03+	2.2463E+03+	2.2334E+03+	2.1003E+03+	2.1608E+03+	2.1634E+03+	2.0716E+03+	2.0814E+03+	**2.0208E+03**
F21	std	4.6318E+01	5.8456E+01	5.4552E+01	2.5774E+00	8.2018E-01	6.8740E+01	5.1407E+01	5.8238E+01	1.2353E+00	3.8702E+01	5.8160E+00
	mean	2.3503E+03+	2.2862E+03+	2.3051E+03+	2.2214E+03+	**2.2021E+03**≈	2.2529E+03+	2.2472E+03+	2.2889E+03+	2.2034E+03≈	2.2323E+03+	2.2028E+03
F22	std	5.0354E+01	1.5102E+01	1.5794E+00	1.4655E+01	2.7529E+00	1.7052E+01	1.8856E+01	1.0768E+01	2.1106E+01	3.4497E+01	2.1061E+01
	mean	2.4007E+03+	2.3065E+03+	2.3038E+03≈	2.3286E+03+	2.3071E+03+	2.3263E+03+	2.3055E+03+	2.3138E+03-	2.3033E+03≈	**2.3007E+03-**	2.3035E+03
F23	std	1.8726E+01	1.1773E+01	1.5584E+01	1.6889E+01	9.4600E+00	7.6708E+00	4.8046E+00	1.3630E+01	8.6088E+00	1.5804E+01	2.4913E+01
	mean	2.6881E+03+	2.6348E+03+	2.6390E+03+	2.6743E+03+	**2.6240E+03-**	2.6399E+03+	2.6453E+03+	2.6295E+03+	2.6311E+03+	2.6323E+03+	2.6254E+03
F24	std	3.8308E+01	1.0714E+02	8.6617E+01	2.5925E+01	1.0863E+01	7.2206E+01	7.4541E+01	2.8141E+01	4.4893E-01	4.9727E+01	1.2859E+02
	mean	2.8477E+03+	2.7028E+03+	2.7436E+03+	2.7768E+03+	2.7510E+03+	2.7327E+03+	2.7453E+03+	2.7555E+03+	2.6315E+03+	2.7429E+03+	**2.5009E+03**
F25	std	1.8781E+01	2.3110E+01	2.2332E+01	1.7302E+01	1.4270E+01	1.7854E+01	1.4517E+01	3.0832E+01	1.0145E+01	1.9577E+01	2.2498E+01
	mean	2.9947E+03+	2.9281E+03+	2.9393E+03+	2.9663E+03+	2.9410E+03+	2.9425E+03+	2.9270E+03+	2.9449E+03+	**2.9048E+03-**	2.9317E+03+	2.9124E+03
F26	std	3.9027E+02	3.6772E+02	4.8108E+02	3.0225E+02	5.8086E+01	2.1605E+01	7.3019E+01	1.2908E+02	1.3051E+02	1.5390E+02	1.8546E+02
	mean	3.6488E+03+	3.0126E+03+	3.2651E+03+	4.0391E+03+	2.8938E+03+	3.0037E+03+	2.9184E+03+	2.9933E+03+	2.8886E+03+	3.0063E+03+	**2.8405E+03**
F27	std	4.6293E+01	7.6630E+00	1.5103E+01	5.1068E+00	2.6569E+00	2.2853E+00	3.0354E+00	2.1109E+01	4.1012E+00	2.8330E+01	2.5866E+01
	mean	3.1966E+03+	3.1057E+03+	3.1044E+03+	3.1192E+03+	3.0947E+03+	3.0946E+03+	3.1069E+03+	3.1046E+03+	3.0999E+03+	3.1138E+03+	**3.0160E+03**
F28	std	1.4572E+02	1.4306E+02	1.1559E+02	4.5088E+01	9.8561E+01	3.9019E+01	3.3314E+01	1.3431E+02	6.5875E+01	1.2712E+02	1.0796E+02
	mean	3.5616E+03+	3.2696E+03+	3.2408E+03+	3.4869E+03+	3.3504E+03+	3.2088E+03+	**3.1832E+03-**	3.3151E+03+	3.1887E+03≈	3.2942E+03+	3.1887E+03
F29	std	4.7016E+01	4.6041E+01	6.7775E+01	4.0642E+01	4.4875E+01	1.6799E+01	2.0087E+01	4.7140E+01	4.0680E+01	6.1308E+01	3.7360E+01
	mean	3.2822E+03+	3.4234E+03+	3.2594E+03+	3.3665E+03+	3.2293E+03+	3.3985E+03+	3.2471E+03+	3.4288E+03+	3.2467E+03+	3.2796E+03+	**3.2128E+03**
F30	std	2.8991E+06	4.6055E+05	4.9493E+05	8.7539E+04	4.4824E+05	2.4950E+05	2.2836E+05	1.1098E+06	2.7645E+05	5.1715E+05	4.8280E+05
	mean	3.7220E+06+	2.3889E+05+	4.3331E+05+	2.4249E+05+	2.7703E+05+	4.5752E+05+	3.5609E+05+	1.2417E+06+	3.1419E+05+	7.8163E+05+	**1.1357E+05**
+/≈/-:		29/0/0	29/0/0	28/1/0	29/0/0	25/1/3	27/0/2	25/0/4	29/0/0	22/3/4	28/0/1	**~**
Avg. rank:		9.65	5.93	6.52	9.21	5.66	6.17	4.90	7.10	3.28	6.03	1.55

Bold values indicate the optimal values, which are consistently used throughout the manuscript.

**Table A3:** Detailed results of optimizers on 30-D CEC2017.

		GA	PSO	SSA	HHO	RIME	SOFA	CPO	HO	SIH	MHO	EBOHO
F1	std	4.5094E+09	8.6490E+08	2.4729E+04	1.0381E+08	2.6227E+06	1.9234E+09	5.2065E+06	1.1881E+08	3.1639E+05	3.3746E+08	1.1809E+05
	mean	4.2177E+09+	6.7105E+08+	2.7457E+05+	2.1849E+08+	4.7605E+06+	6.4917E+09+	1.2912E+07+	1.8982E+08+	1.2698E+06+	2.3695E+08+	**6.0369E+04**
F3	std	6.4421E+04	1.3485E+04	8.2604E+03	7.3397E+03	8.1684E+03	1.2063E+04	6.8504E+03	7.3728E+03	3.2671E+03	6.0559E+03	5.8240E+03
	mean	2.7470E+05+	4.0119E+04+	5.7717E+04+	3.9519E+04+	2.4140E+04+	3.9267E+04+	5.7730E+04+	5.0748E+04+	3.7928E+04+	4.6927E+04+	**1.0922E+04**
F4	std	3.9713E+02	5.3110E+01	1.0459E+01	8.3017E+01	1.9104E+01	2.8444E+02	1.5239E+01	4.9919E+01	2.7745E+01	4.2643E+01	4.7084E+01
	mean	1.1527E+03+	5.4118E+02+	5.1436E+02+	6.5910E+02+	5.0897E+02+	9.0720E+02+	5.4966E+02+	6.3438E+02+	5.3633E+02+	6.1836E+02+	**5.0713E+02**
F5	std	5.1878E+01	3.4578E+01	5.0187E+01	1.8812E+01	2.2160E+01	3.1920E+01	1.1054E+01	2.2935E+01	3.1297E+01	4.6722E+01	2.2725E+01
	mean	8.3050E+02+	7.2294E+02+	7.2754E+02+	7.5149E+02+	7.8495E+02+	7.9951E+02+	7.0636E+02+	7.1836E+02+	7.0236E+02≈	7.1913E+02+	**7.0226E+02**
F6	std	7.6192E+00	7.3883E+00	9.5844E+00	2.7141E+00	1.2909E+00	8.6014E+00	1.1570E+00	8.4366E+00	7.4097E+00	7.8538E+00	5.7738E+00
	mean	6.9431E+02+	6.2841E+02+	6.4302E+02+	6.6579E+02+	6.2471E+02+	6.5815E+02+	6.1726E+02+	6.5556E+02+	6.5221E+02+	6.6035E+02+	**6.1111E+02**
F7	std	1.0898E+02	3.7133E+01	1.2697E+02	6.1894E+01	3.2926E+01	7.9243E+01	8.2251E+00	7.2758E+01	3.3018E+01	6.9273E+01	4.0058E+01
	mean	1.2142E+03+	1.1278E+03+	1.1972E+03+	1.3250E+03+	**8.4693E+02-**	1.1625E+03+	9.3516E+02+	1.1269E+03+	9.8799E+02+	1.1234E+03+	9.1078E+02
F8	std	3.2442E+01	2.4383E+01	2.9732E+01	2.8008E+01	2.5636E+01	3.2531E+01	1.1897E+01	2.5593E+01	2.5021E+01	2.8171E+01	1.4170E+01
	mean	1.0867E+03+	8.9830E+02+	9.7743E+02+	9.6885E+02+	8.8938E+02+	1.1144E+03+	9.9678E+02+	9.6086E+02+	9.3541E+02+	9.5753E+02+	**8.8420E+02**
F9	std	1.0100E+03	7.5074E+02	6.4004E+02	5.7783E+02	5.6915E+02	2.2423E+03	1.1491E+02	1.1056E+03	9.0389E+02	1.1275E+03	1.0930E+03
	mean	6.0209E+03+	4.7403E+03+	5.0725E+03+	8.1911E+03+	6.7261E+03+	8.2607E+03+	6.1663E+03+	5.7249E+03+	5.1915E+03+	5.5803E+03+	**4.6450E+03**
F10	std	6.3461E+02	7.1603E+02	5.8105E+02	5.0275E+02	5.2407E+02	9.9491E+02	4.1193E+02	5.6491E+02	5.3019E+02	3.1375E+02	6.5921E+02
	mean	7.5616E+03+	5.5469E+03+	5.2919E+03+	5.9436E+03+	5.2754E+03+	8.1644E+03+	7.7428E+03+	5.3332E+03+	5.2132E+03+	5.7123E+03+	**5.1184E+03**
F11	std	6.4130E+03	3.6422E+01	6.9437E+01	9.1317E+01	7.2732E+01	7.1752E+01	3.1055E+01	1.6587E+02	5.2271E+01	1.2917E+02	4.9756E+01
	mean	1.2465E+04+	1.2848E+03+	1.2731E+03+	1.3683E+03+	1.3044E+03+	1.6031E+03+	1.3543E+03+	1.7173E+03+	1.3098E+03+	1.6440E+03+	**1.2577E+03**
F12	std	7.1735E+07	4.4080E+06	1.2444E+06	1.3708E+07	1.0781E+07	3.8454E+07	1.7722E+06	3.3247E+07	1.4473E+07	1.4146E+08	9.4939E+06
	mean	8.3024E+07+	1.8403E+06+	6.3633E+06+	1.8546E+07+	2.1019E+07+	8.1928E+07+	3.4341E+06+	8.1722E+07+	1.7512E+07+	1.2358E+08+	**1.4117E+06**
F13	std	2.3081E+07	2.2548E+07	1.9895E+04	2.7572E+05	6.8987E+04	6.3905E+05	1.0814E+05	7.9418E+04	1.9365E+04	6.5088E+04	2.4161E+04
	mean	1.5562E+07+	7.6354E+06+	6.1145E+04+	2.0095E+04≈	1.0408E+05+	1.0158E+06+	1.7801E+05+	1.2408E+05+	5.6734E+04+	1.1360E+05+	**2.0095E+04**
F14	std	1.7471E+06	3.3773E+04	4.2602E+04	7.8990E+04	3.1391E+04	1.1000E+02	2.2906E+03	3.5532E+05	2.3957E+04	1.5735E+05	6.3640E+04
	mean	2.7224E+06+	3.7595E+04+	5.3210E+04+	1.0935E+05+	4.4203E+04+	**1.9637E+03-**	5.3816E+03+	2.8075E+05+	2.7111E+04+	1.3654E+05+	3.1104E+03
F15	std	4.0468E+05	1.9069E+04	1.1102E+04	2.4599E+04	1.5336E+04	2.8710E+04	8.4299E+03	1.7564E+04	2.0045E+03	4.6468E+04	1.6150E+04
	mean	3.1125E+05+	1.4955E+04+	1.1833E+04+	8.8827E+04+	2.5203E+04+	5.8089E+04+	2.0538E+04+	2.9038E+04+	1.3133E+04+	5.0729E+04+	**1.1222E+04**
F16	std	2.3125E+02	3.1610E+02	2.4084E+02	2.0263E+02	1.7014E+02	2.6374E+02	2.0966E+02	2.9456E+02	1.4065E+02	3.2128E+02	4.6886E+02
	mean	3.1203E+03+	2.6708E+03+	2.8355E+03+	3.3507E+03+	2.6126E+03+	3.7030E+03+	3.1726E+03+	3.3372E+03+	2.9604E+03+	3.4397E+03+	**2.2574E+03**
F17	std	2.0150E+02	1.5685E+02	2.0342E+02	2.7378E+02	2.1764E+02	1.3706E+02	7.9010E+01	2.0770E+02	1.2786E+02	2.4423E+02	1.7273E+02
	mean	2.3878E+03+	2.0996E+03+	2.4298E+03+	2.8321E+03+	2.1323E+03+	2.6476E+03+	2.1581E+03+	2.4435E+03+	**2.0924E+03-**	2.1841E+03+	2.0945E+03
F18	std	5.3211E+06	2.0087E+05	5.8338E+05	4.9053E+05	8.3672E+05	9.1944E+04	5.8015E+04	7.4319E+05	3.9397E+04	7.5899E+05	1.0198E+05
	mean	8.5130E+06+	2.3998E+05+	6.3347E+05+	6.0230E+05+	8.6731E+05+	1.6207E+05+	2.6333E+05+	7.3029E+05+	1.5636E+05+	9.5644E+05+	**9.6647E+04**
F19	std	1.1674E+06	1.8617E+04	1.5804E+04	1.0117E+06	1.3136E+04	2.1048E+05	9.7715E+03	1.9193E+06	6.7901E+04	1.9452E+06	3.1880E+04
	mean	1.3626E+06+	2.1718E+04+	3.4223E+05+	9.3030E+05+	1.8415E+04-	2.5086E+05+	**1.7704E+04-**	2.9936E+06+	9.3183E+04+	3.6588E+06+	1.9250E+04
F20	std	2.0624E+02	1.7769E+02	2.1202E+02	1.3870E+02	1.3405E+02	8.7669E+01	1.1089E+02	1.0182E+02	1.2943E+02	1.4568E+02	9.0161E+01
	mean	2.8345E+03+	2.5236E+03+	2.7035E+03+	2.5960E+03+	2.3745E+03+	2.8154E+03+	2.5502E+03+	2.4673E+03+	2.4345E+03+	2.5232E+03+	**2.3206E+03**
F21	std	4.2420E+01	3.5723E+01	4.8503E+01	4.0646E+01	1.8268E+01	3.6525E+01	1.7269E+01	4.3664E+01	1.1499E+02	3.2029E+01	2.7988E+01
	mean	2.6868E+03+	2.4287E+03+	2.4957E+03+	2.5991E+03+	2.3863E+03+	2.5935E+03+	2.4915E+03+	2.5213E+03+	2.4162E+03+	2.5013E+03+	**2.2140E+03**
F22	std	2.3152E+03	1.8824E+03	2.1629E+03	1.3823E+03	1.6231E+03	3.0394E+03	2.2495E+00	1.7382E+03	4.2335E+00	2.4735E+03	4.1423E+00
	mean	7.2169E+03+	3.8958E+03+	5.3140E+03+	6.3325E+03+	3.2917E+03+	5.2167E+03+	2.3220E+03+	3.8044E+03+	2.3149E+03+	4.5309E+03+	**2.3118E+03**
F23	std	4.8627E+01	6.2874E+01	5.7343E+01	7.9589E+01	2.8629E+01	2.3470E+01	1.4619E+01	8.6147E+01	8.1652E+01	4.7787E+01	1.1145E+02
	mean	3.1997E+03+	2.9139E+03+	2.9384E+03+	3.2236E+03+	**2.7533E+03-**	2.9332E+03+	2.8594E+03+	2.9645E+03+	2.9663E+03+	2.9652E+03+	2.8092E+03
F24	std	7.0904E+01	1.0004E+02	8.6975E+01	1.5237E+02	3.6287E+01	1.7034E+01	1.1943E+01	8.6219E+01	2.2713E+02	1.1375E+02	9.4172E+01
	mean	3.3349E+03+	3.0506E+03+	3.0783E+03+	3.5520E+03+	2.9307E+03+	3.0756E+03+	3.0364E+03+	3.1235E+03+	3.1513E+03+	3.1863E+03+	**2.8925E+03**
F25	std	3.0677E+02	2.4210E+01	1.1480E+01	4.0537E+01	3.1748E+01	2.6459E+02	1.3651E+01	2.1779E+01	2.9743E+01	4.3871E+01	3.2670E+01
	mean	3.9214E+03+	2.9123E+03+	2.8986E+03+	2.9783E+03+	2.9228E+03+	3.2844E+03+	2.9246E+03+	3.0169E+03+	2.9401E+03+	3.0279E+03+	**2.8303E+03**
F26	std	3.2591E+02	1.0188E+03	1.2556E+03	5.5550E+02	2.4023E+02	1.2041E+03	1.2426E+03	1.6309E+03	2.9042E+02	6.3223E+02	9.4792E+02
	mean	7.0635E+03+	5.0742E+03+	6.0650E+03+	8.1781E+03+	4.6581E+03+	5.8377E+03+	4.9113E+03+	6.9250E+03+	3.2766E+03+	6.8085E+03+	**3.1422E+03**
F27	std	1.0119E+02	4.7970E+01	7.6842E+01	1.3869E+02	2.2201E+01	1.1446E+01	1.4402E+01	1.1772E+02	6.2087E+01	1.2173E+02	1.4982E+02
	mean	3.7119E+03+	3.2864E+03+	3.2857E+03≈	3.5657E+03+	3.2340E+03-	**3.2296E+03-**	3.3280E+03+	3.4704E+03+	3.3708E+03+	3.4569E+03+	3.2850E+03
F28	std	3.6362E+02	2.9824E+02	1.4502E+01	8.3656E+01	6.3566E+01	5.4030E+02	2.4704E+01	6.4643E+01	3.2774E+01	5.8231E+01	2.9612E+01
	mean	4.5934E+03+	3.3899E+03+	**3.2600E+03-**	3.4369E+03+	3.3049E+03+	3.7164E+03+	3.2943E+03+	3.3820E+03+	3.2759E+03+	3.4049E+03+	3.2663E+03
F29	std	2.2243E+02	1.8717E+02	4.0824E+02	3.2113E+02	2.1188E+02	2.2270E+02	1.2082E+02	4.0268E+02	2.3149E+02	2.7398E+02	2.5263E+02
	mean	4.7292E+03+	3.9923E+03+	4.2291E+03+	4.5321E+03+	**3.8048E+03-**	4.3531E+03+	4.1634E+03+	4.7933E+03+	4.3867E+03+	4.7384E+03+	3.9845E+03
F30	std	3.7543E+06	1.0160E+04	1.5606E+04	1.5685E+06	4.4867E+05	4.6247E+05	1.4272E+05	1.4895E+07	1.0719E+06	5.4535E+06	5.0070E+05
	mean	4.1815E+06+	2.3055E+04+	5.1663E+05+	5.9471E+06+	6.1862E+05+	5.5030E+05+	4.0560E+05+	2.1049E+07+	1.4691E+06+	1.1010E+07+	**1.9859E+04**
+/≈/-:		29/0/0	29/0/0	27/1/1	28/1/0	24/0/5	27/0/2	28/0/1	29/0/0	27/1/1	29/0/0	~
Avg. rank:		10.00	4.66	5.31	8.48	3.86	7.76	5.03	7.52	4.21	7.83	1.34

Bold values indicate the optimal values, which are consistently used throughout the manuscript.

**Table A4:** Detailed results of optimizers on 50-D CEC2017.

		GA	PSO	SSA	HHO	RIME	SOFA	CPO	HO	SIH	MHO	EBOHO
F1	std	1.2843E+10	1.2579E+09	1.1412E+05	1.3423E+08	3.5670E+06	8.8197E+09	7.4080E+06	1.0946E+09	5.2181E+05	9.2781E+08	8.7197E+05
	mean	4.3951E+10+	1.4648E+09+	6.3817E+05+	3.0523E+08+	1.3629E+07+	1.8716E+10+	2.6822E+07+	2.1098E+09+	3.0161E+06+	1.8740E+09+	**3.6977E+05**
F3	std	1.0579E+05	2.7178E+04	2.9159E+04	8.3769E+03	2.6917E+04	2.9015E+04	2.2474E+04	1.3159E+04	3.8337E+03	1.4271E+04	1.2502E+04
	mean	4.2613E+05+	1.2642E+05+	1.9982E+05+	8.4666E+04+	8.5606E+04+	1.0423E+05+	1.6649E+05+	1.0624E+05+	4.2080E+04+	1.1222E+05+	**3.4102E+04**
F4	std	1.6704E+03	1.7575E+02	6.7693E+01	9.9730E+01	6.2536E+01	8.6876E+02	3.7483E+01	1.7632E+02	3.6577E+01	1.3095E+02	4.3429E+01
	mean	4.1147E+03+	7.9821E+02+	6.6949E+02+	8.4156E+02+	6.4329E+02+	2.0312E+03+	6.5803E+02+	1.2477E+03+	6.8486E+02+	1.1968E+03+	**6.2233E+02**
F5	std	4.0066E+01	7.1219E+01	2.5926E+01	3.6008E+01	4.9175E+01	5.7411E+01	2.2828E+01	5.0901E+01	2.8832E+01	3.5070E+01	2.0901E+01
	mean	1.1594E+03+	8.6882E+02+	8.5908E+02+	9.1992E+02+	9.9981E+02+	1.0901E+03+	8.9299E+02+	8.9063E+02+	8.9678E+02+	8.5161E+02≈	**8.5077E+02**
F6	std	1.0894E+01	9.4610E+00	7.9786E+00	4.4740E+00	4.4504E+00	8.4798E+00	7.4201E-01	5.6443E+00	7.1915E+00	2.9298E+00	5.4184E+00
	mean	7.1631E+02+	7.4313E+02+	6.9032E+02+	6.7386E+02+	6.9570E+02+	6.7216E+02+	6.7555E+02+	6.6872E+02+	**6.6471E+02≈**	6.7006E+02+	6.6472E+02
F7	std	2.6708E+02	1.1303E+02	1.0301E+02	6.7887E+01	5.3980E+01	6.2386E+01	2.6067E+01	7.7513E+01	9.7169E+01	1.0302E+02	1.1601E+02
	mean	2.2505E+03+	1.1215E+03+	1.7020E+03+	1.8180E+03+	**1.0118E+03≈**	1.6084E+03+	1.1691E+03+	1.6399E+03+	1.3505E+03+	1.5588E+03+	1.0128E+03
F8	std	5.6959E+01	4.7839E+01	2.7356E+01	2.9656E+01	5.2573E+01	5.7956E+01	1.0048E+01	4.2579E+01	3.9238E+01	4.3060E+01	1.9134E+01
	mean	1.3959E+03+	1.0643E+03+	1.2026E+03+	1.2225E+03+	**9.7507E+02-**	1.4073E+03+	1.1958E+03+	1.1295E+03+	1.1345E+03+	1.1287E+03+	9.8739E+02
F9	std	3.5228E+03	1.9093E+03	1.8729E+03	1.8086E+03	1.8918E+03	7.4904E+03	6.8956E+02	1.8820E+03	2.7383E+03	2.1527E+03	3.5446E+03
	mean	1.6430E+04+	1.0209E+04+	1.3551E+04+	2.4984E+04+	4.4418E+03+	3.0373E+04+	2.6845E+03+	1.7237E+04+	1.5044E+04+	1.7896E+04+	**2.5031E+03**
F10	std	7.5950E+02	8.0934E+02	8.3191E+02	9.2683E+02	7.6380E+02	9.1187E+02	4.0954E+02	1.1707E+03	1.0402E+03	1.1174E+03	9.7283E+02
	mean	1.2539E+04+	8.6065E+03+	8.8146E+03+	1.0584E+04+	**7.0342E+03-**	1.4494E+04+	1.3210E+04+	8.3898E+03+	8.3586E+03+	8.9234E+03+	7.5299E+03
F11	std	1.8599E+04	7.4071E+01	6.7418E+01	1.2718E+02	1.0774E+02	2.9031E+02	3.3493E+01	4.9898E+02	1.1527E+02	2.7503E+02	5.6386E+01
	mean	3.5127E+04+	1.4458E+03+	**1.4005E+03-**	1.6678E+03+	1.5694E+03+	2.2986E+03+	1.5505E+03+	2.5195E+03+	1.6424E+03+	2.3162E+03+	1.4139E+03
F12	std	3.4046E+09	6.2008E+08	4.6541E+06	1.0769E+08	4.3044E+07	3.6472E+08	3.0957E+06	2.2886E+08	1.4431E+07	1.7616E+08	4.9763E+07
	mean	4.2984E+09+	2.9174E+08+	1.1394E+07+	1.9506E+08+	6.8176E+07+	8.1877E+08+	5.7090E+07+	3.8017E+08+	3.7707E+07+	3.9871E+08+	**9.8760E+06**
F13	std	1.2981E+08	1.9935E+08	2.3969E+04	9.1078E+05	1.2541E+05	7.5983E+06	1.1718E+04	1.2482E+07	4.1829E+04	2.8913E+05	3.8577E+04
	mean	1.4470E+08+	6.9572E+07+	9.3352E+04+	3.4133E+06+	2.8069E+05+	1.6020E+07+	**2.6526E+04-**	4.5778E+06+	1.3968E+05+	3.0277E+05+	8.8808E+04
F14	std	1.1921E+07	2.6661E+05	2.8314E+05	1.1105E+06	2.6789E+05	4.8790E+04	4.7026E+04	1.2271E+06	1.5252E+05	1.6109E+06	4.6482E+04
	mean	1.7726E+07+	6.6858E+03+	5.3858E+03+	3.7735E+05+	9.4178E+04+	1.0949E+05+	1.6119E+04+	3.7237E+04+	7.3560E+03+	3.5121E+04+	**1.8945E+04**
F15	std	1.5647E+07	1.2319E+05	2.1092E+05	7.0929E+05	9.6544E+04	2.4115E+05	2.6894E+04	4.8871E+04	2.8859E+04	5.3986E+04	2.5300E+04
	mean	4.4089E+02+	5.5001E+02+	4.1302E+02+	5.7942E+02+	3.4377E+02+	3.5386E+02+	3.1612E+02+	7.7144E+02+	3.4741E+02+	5.7257E+02+	**6.3559E+02**
F16	std	5.0912E+04	3.1157E+04	3.9035E+03	4.0412E+03	3.3718E+04	5.3521E+03	4.2601E+03	4.7051E+03	3.9059E+03	4.7877E+03	3.9044E+03
	mean	1.1921E+07+	2.6661E+05+	**2.8314E+05≈**	1.1105E+06+	2.6789E+05+	4.8790E+04+	4.7026E+04+	1.2271E+06+	1.5252E+05+	1.6109E+06+	4.6482E+04
F17	std	5.1446E+02	3.0094E+02	3.9262E+02	3.6113E+02	3.2424E+02	2.8740E+02	1.8103E+02	3.2642E+02	2.2378E+02	2.2391E+02	3.6713E+02
	mean	3.8745E+03+	3.3023E+03+	3.7914E+03+	3.6840E+03+	3.2044E+03+	4.5270E+03+	3.3587E+03+	3.6978E+03+	**3.0691E+03-**	3.6930E+03+	3.1152E+03
F18	std	1.9307E+07	4.4104E+05	1.3359E+06	5.6975E+06	2.7537E+06	4.7081E+05	6.0497E+05	2.6089E+06	6.6373E+05	1.7324E+06	7.4233E+05
	mean	2.7518E+07+	6.0243E+05+	2.2010E+06+	6.1453E+06+	3.8883E+06+	1.0132E+06+	1.3102E+06+	3.3938E+06+	1.2776E+06+	2.0854E+06+	**5.8216E+05**
F19	std	5.2751E+06	1.2141E+04	9.3459E+03	1.2015E+06	2.0401E+05	7.4211E+05	4.7253E+03	5.4043E+06	1.6131E+04	7.4987E+06	1.9563E+04
	mean	9.5937E+06+	5.9504E+04+	4.9994E+04+	1.5693E+06+	1.7789E+05+	8.9216E+05+	**2.3426E+04-**	4.4081E+06+	4.1721E+04+	1.1475E+07+	3.3830E+04
F20	std	2.3358E+02	3.1574E+02	3.1057E+02	2.4666E+02	3.0272E+02	1.6042E+02	1.8831E+02	2.1444E+02	1.1851E+02	2.0714E+02	1.7163E+02
	mean	3.7712E+03+	3.1757E+03+	3.6937E+03+	3.6966E+03+	3.1826E+03+	4.1418E+03+	3.5383E+03+	3.1104E+03+	**2.9554E+03-**	3.1401E+03+	3.0253E+03
F21	std	5.4321E+01	5.0459E+01	1.1729E+02	2.1763E+01	3.7564E+01	4.6975E+01	2.0234E+01	6.3394E+01	5.7178E+01	8.3872E+01	6.6870E+01
	mean	3.1111E+03+	2.6295E+03+	2.7471E+03+	2.7418E+03+	2.4756E+03+	2.9041E+03+	2.6821E+03+	2.7387E+03+	2.7185E+03+	2.7253E+03+	**2.3342E+03**
F22	std	1.1789E+03	1.0064E+03	8.4711E+02	1.2365E+03	8.6080E+02	4.5208E+02	6.4744E+03	8.4201E+02	1.0799E+03	1.3033E+03	1.1244E+03
	mean	1.5188E+04+	1.2222E+04+	1.0247E+04+	1.1531E+04+	**8.9868E+03-**	1.6185E+04+	9.8845E+03+	1.0382E+04+	1.0663E+04+	1.1241E+04+	9.1833E+03
F23	std	1.5480E+02	8.3311E+01	1.5677E+02	1.2801E+02	4.7812E+01	6.6851E+01	2.7728E+01	9.6413E+01	9.5019E+01	1.1875E+02	1.4310E+02
	mean	3.7217E+03+	3.2548E+03+	3.3523E+03+	3.8095E+03+	3.5278E+03+	3.3605E+03+	3.1356E+03+	3.4069E+03+	3.5089E+03+	3.4421E+03+	**3.0636E+03**
F24	std	1.4753E+02	1.3536E+02	9.1719E+01	2.1567E+02	4.9167E+01	3.6642E+01	3.0771E+01	1.7843E+02	1.0888E+02	1.2558E+02	1.7378E+02
	mean	4.1963E+03+	3.4547E+03+	3.4746E+03+	4.3300E+03+	3.6785E+03+	3.4447E+03+	3.3043E+03+	3.7322E+03+	3.4195E+03+	3.6707E+03+	**3.1176E+03**
F25	std	1.1500E+03	2.6324E+02	2.6460E+01	3.5475E+01	3.4091E+01	2.7952E+02	2.5080E+01	1.8924E+02	3.7033E+01	2.0807E+02	2.7962E+01
	mean	7.9137E+03+	3.2946E+03+	3.3921E+03+	3.2762E+03+	3.1255E+03≈	4.4556E+03+	3.1844E+03+	3.6068E+03+	3.1807E+03+	3.4266E+03+	**3.1245E+03**
F26	std	9.2313E+02	1.3397E+03	3.0604E+03	2.3227E+03	5.2834E+02	2.3459E+03	1.8879E+03	1.3611E+03	4.0904E+02	2.6456E+03	1.3138E+03
	mean	1.1861E+04+	8.9793E+03+	7.8258E+03+	9.6505E+03+	5.9176E+03+	8.2170E+03+	7.1316E+03+	1.1120E+04+	**3.8115E+03-**	1.1872E+04+	4.4358E+03
F27	std	2.6169E+02	1.1824E+02	1.6762E+02	2.9566E+02	8.6827E+01	8.1875E+01	7.6287E+01	2.5620E+02	1.6254E+02	2.0095E+02	4.6162E+02
	mean	4.7806E+03+	3.6523E+03+	3.6883E+03+	4.2954E+03+	3.5733E+03+	3.4640E+03+	3.7240E+03+	4.1703E+03+	3.9028E+03+	4.3160E+03+	**3.3987E+03**
F28	std	1.0280E+03	5.6551E+02	4.4929E+01	8.9293E+01	1.5794E+01	3.2430E+02	3.9597E+01	2.6587E+02	4.3541E+01	3.6185E+02	4.0566E+01
	mean	8.0222E+03+	3.7848E+03+	**3.3635E+03-**	3.7502E+03+	3.3896E+03-	4.2422E+03+	3.5011E+03+	4.3322E+03+	3.4369E+03+	4.3188E+03+	3.4134E+03
F29	std	5.8058E+02	3.4229E+02	4.0758E+02	8.1609E+02	2.4683E+02	3.2971E+02	1.5420E+02	5.5228E+02	3.8152E+02	6.4088E+02	5.5798E+02
	mean	6.2681E+03+	5.0424E+03+	5.0295E+03+	6.2830E+03+	**4.7808E+03-**	5.9905E+03+	5.1437E+03+	7.6092E+03+	5.6705E+03+	7.7621E+03+	4.8692E+03
F30	std	2.1361E+08	4.6575E+06	3.0866E+05	1.0692E+07	1.0731E+07	6.8335E+06	3.2899E+06	7.5207E+07	1.9748E+07	7.2010E+07	1.0786E+07
	mean	3.9531E+08+	8.6710E+06+	2.6032E+07+	3.0537E+07+	2.5622E+07+	1.9329E+07+	1.5372E+07+	2.1608E+08+	9.9610E+07+	1.5674E+08+	**1.4591E+06**
+/≈/-:		29/0/0	29/0/0	26/1/2	29/0/0	22/1/6	29/0/0	27/0/2	29/0/0	25/1/3	28/1/0	~
Avg. rank:		10.38	5.45	5.35	7.72	4.52	7.79	4.38	7.34	4.17	7.38	1.52

Bold values indicate the optimal values, which are consistently used throughout the manuscript.

**Table A5:** Detailed results of optimizers on 100-D CEC2017.

		GA	PSO	SSA	HHO	RIME	SOFA	CPO	HO	SIH	MHO	EBOHO
F1	std	4.5035E+10	6.3169E+09	5.9194E+05	8.9276E+07	1.8199E+07	9.7372E+09	8.5674E+06	2.2591E+09	3.0732E+06	3.5189E+09	3.4627E+05
	mean	2.6576E+11+	1.7911E+10+	1.7632E+06+	8.6635E+08+	6.1790E+07+	5.0851E+10+	3.0802E+07+	1.3087E+10+	1.0262E+07+	1.3111E+10+	**1.3371E+06**
F3	std	1.8713E+05	6.4096E+04	4.2611E+04	3.2827E+04	7.3607E+04	5.6878E+04	3.3147E+04	1.6957E+04	1.9577E+04	1.3309E+04	2.4779E+04
	mean	9.3142E+05+	3.7586E+05+	3.7136E+05+	2.3181E+05+	3.3474E+05+	2.2655E+05+	3.5988E+05+	2.7393E+05+	2.0886E+05+	2.8545E+05+	**1.4139E+05**
F4	std	8.7589E+03	1.0787E+03	3.3468E+01	1.0241E+02	8.5558E+01	1.7943E+03	3.2246E+01	4.5612E+02	7.5517E+01	3.2223E+02	5.3673E+01
	mean	3.2014E+04+	3.2422E+03+	7.5260E+02+	1.2446E+03+	8.6076E+02+	6.3561E+03+	8.9351E+02+	3.3752E+03+	9.7528E+02+	3.3213E+03+	**7.4873E+02**
F5	std	1.0296E+02	6.2864E+01	4.0160E+01	5.2032E+01	6.5421E+01	9.7495E+01	3.9130E+01	5.7521E+01	3.7321E+01	4.9506E+01	2.3681E+01
	mean	2.2210E+03+	1.2254E+03+	1.3742E+03+	1.4857E+03+	**9.5731E+02-**	1.8999E+03+	1.4937E+03+	1.3475E+03+	1.3360E+03+	1.3540E+03+	9.6527E+02
F6	std	5.6686E+00	6.5695E+00	1.9308E+00	3.0823E+00	7.0627E+00	9.2847E+00	1.0444E+00	4.3678E+00	3.5052E+00	2.8017E+00	5.6928E+00
	mean	7.3555E+02+	6.9553E+02+	6.6378E+02+	6.8573E+02+	6.2669E+02-	6.9790E+02+	**6.0534E+02-**	6.6982E+02+	6.7017E+02+	6.6995E+02+	6.5971E+02
F7	std	8.3210E+02	1.4945E+02	1.7336E+02	1.0794E+02	1.3653E+02	8.6145E+01	6.0699E+01	1.1361E+02	3.1078E+02	1.7553E+02	1.2624E+02
	mean	6.9050E+03+	4.3936E+03+	3.2230E+03+	3.6731E+03+	3.5049E+03+	2.8753E+03+	3.8210E+03+	3.0600E+03+	**2.6930E+03-**	3.0904E+03+	2.7799E+03
F8	std	1.0369E+02	6.3343E+01	6.6177E+01	3.3157E+01	5.1249E+01	9.5544E+01	3.5646E+01	7.0250E+01	8.7328E+01	7.5199E+01	5.6557E+01
	mean	2.5782E+03+	1.6089E+03+	1.8214E+03+	1.9526E+03+	1.2038E+03+	2.2713E+03+	1.7758E+03+	1.7492E+03+	1.7417E+03+	1.8156E+03+	**1.1485E+03**
F9	std	1.1138E+04	3.9631E+03	2.6162E+02	7.7881E+03	6.3853E+03	6.5532E+03	8.7337E+03	2.2302E+03	1.7801E+03	2.4223E+03	1.2477E+03
	mean	8.5288E+04+	2.2399E+04+	2.3636E+04+	5.3632E+04+	2.0010E+04+	8.1110E+04+	1.9348E+04+	3.1711E+04+	3.0347E+04+	3.2241E+04+	**1.8377E+04**
F10	std	1.6645E+03	1.6239E+03	1.5230E+03	1.4854E+03	1.3780E+03	1.1283E+03	3.7024E+02	1.2912E+03	1.2784E+03	3.0825E+03	2.5024E+03
	mean	2.9426E+04+	1.9227E+04+	1.9223E+04+	2.1339E+04+	**1.6193E+04-**	3.0963E+04+	2.9053E+04+	1.8632E+04+	1.6663E+04-	1.9119E+04+	1.7045E+04
F11	std	5.4645E+04	9.6793E+02	4.6765E+03	2.2096E+03	4.7781E+02	6.5219E+03	5.0807E+03	8.5766E+03	4.9154E+02	1.0424E+04	3.5732E+03
	mean	2.4992E+05+	4.8526E+04+	2.8184E+04+	1.0381E+04+	4.6465E+03+	1.9515E+04+	2.8955E+04+	5.9608E+04+	5.0866E+03+	5.4615E+04+	**4.3574E+03**
F12	std	1.9576E+10	2.5177E+09	1.3756E+07	2.2866E+08	2.9535E+08	2.0172E+09	7.6196E+06	6.7561E+08	6.2252E+07	7.4967E+08	2.6782E+07
	mean	4.7061E+10+	4.8687E+09+	4.3124E+07+	5.9209E+08+	6.3757E+08+	5.4360E+09+	3.1017E+07+	1.8945E+09+	3.4139E+08+	1.4375E+09+	**2.9980E+07**
F13	std	4.2822E+09	2.6619E+08	1.0112E+04	1.8443E+06	3.7983E+04	3.3231E+08	1.7858E+03	1.6683E+06	2.8285E+04	4.2002E+04	3.6435E+04
	mean	5.3894E+09+	2.0580E+08+	1.7169E+04+	8.8457E+06+	2.0356E+05+	2.1360E+08+	4.8973E+03+	6.6796E+05+	6.8733E+04+	9.4172E+04+	**4.3171E+03**
F14	std	2.9677E+07	1.1729E+06	2.4020E+05	5.2995E+05	1.4589E+06	2.0893E+05	1.6223E+05	1.6838E+06	8.5335E+05	1.6370E+06	3.3435E+05
	mean	6.1308E+07+	1.1629E+06+	7.7553E+05+	2.6946E+06+	2.9054E+06+	**1.9826E+05-**	5.5873E+05+	4.3739E+06+	1.1497E+06+	3.9278E+06+	3.1539E+05
F15	std	2.2565E+07	3.6897E+08	4.5857E+03	5.7602E+05	3.4818E+04	1.1077E+06	1.3305E+03	6.1826E+04	7.8623E+03	8.8923E+04	1.1362E+04
	mean	2.6696E+07+	1.7477E+08+	7.7692E+03+	2.1452E+06+	1.1059E+05+	1.9667E+06+	2.8718E+03+	9.4311E+04+	3.8106E+04+	9.0531E+04+	**2.8393E+03**
F16	std	9.5969E+02	5.2995E+02	7.9100E+02	7.5077E+02	6.3957E+02	5.6408E+02	3.8831E+02	9.4554E+02	7.5942E+02	1.3634E+03	7.3040E+02
	mean	1.0856E+04+	6.3115E+03+	6.2142E+03+	8.4562E+03+	6.9620E+03+	1.1075E+04+	9.1636E+03+	8.8743E+03+	7.8782E+03+	9.9435E+03+	**6.1773E+03**
F17	std	1.3552E+03	6.6173E+02	7.4205E+02	2.5703E+02	5.1723E+02	6.9823E+02	2.3936E+02	7.2107E+02	2.5023E+02	7.3714E+02	7.2765E+02
	mean	8.0648E+03+	5.4926E+03-	6.2410E+03+	6.3325E+03+	5.6675E+03+	7.8192E+03+	6.1911E+03+	7.3789E+03+	**5.2875E+03-**	7.1251E+03+	5.5417E+03
F18	std	2.3786E+07	5.7193E+05	5.6221E+05	8.3614E+05	2.2976E+06	4.0842E+05	1.0370E+06	7.6895E+05	3.3237E+05	1.9121E+06	4.2757E+05
	mean	4.1216E+07+	1.0994E+06+	1.5296E+06+	3.8847E+06+	5.5777E+06+	9.6096E+05+	1.6927E+06+	2.3567E+06+	1.1237E+06+	3.3228E+06+	**6.4722E+05**
F19	std	1.3038E+08	2.7554E+08	8.4127E+03	2.4050E+06	2.7572E+06	1.2850E+07	1.7070E+03	1.5703E+07	2.9690E+05	1.3333E+07	2.2420E+05
	mean	1.5171E+08+	1.7595E+08+	8.9267E+05+	7.1779E+06+	4.4032E+06+	1.9834E+07+	3.8577E+05+	2.3134E+07+	4.7516E+05+	2.5519E+07+	**2.5429E+05**
F20	std	3.3308E+02	5.9511E+02	4.0872E+02	4.9786E+02	4.9843E+02	3.0942E+02	3.0596E+02	2.3210E+02	2.8094E+02	2.6055E+02	2.1320E+02
	mean	6.9250E+03+	5.5536E+03+	5.9966E+03+	6.2815E+03+	5.5172E+03+	7.6219E+03+	6.5717E+03+	6.0189E+03+	**4.8624E+03-**	5.1883E+03+	5.0707E+03
F21	std	2.5656E+02	1.3330E+02	2.9406E+02	1.0057E+02	9.9559E+01	7.8166E+01	2.4983E+01	1.3320E+02	2.0615E+02	9.6806E+01	1.6397E+02
	mean	4.6210E+03+	3.3425E+03+	3.5893E+03+	4.0294E+03+	**2.8181E+03-**	3.8197E+03+	3.2267E+03+	3.5738E+03+	3.5546E+03+	3.5615E+03+	2.8929E+03
F22	std	1.3921E+03	1.4257E+03	1.0058E+03	1.3821E+03	1.8905E+03	6.2664E+03	6.1909E+02	2.1622E+03	9.2069E+02	9.7210E+02	1.2635E+03
	mean	3.3252E+04+	2.6693E+04+	2.9479E+04+	2.4669E+04+	2.2312E+04+	3.1442E+04+	3.1630E+04+	2.1480E+04+	2.1208E+04+	2.2412E+04+	**2.1148E+04**
F23	std	2.4338E+02	1.9461E+02	2.3797E+02	2.1081E+02	8.9467E+01	1.5370E+02	2.9373E+01	3.6825E+02	2.4614E+02	2.0655E+02	2.2878E+02
	mean	5.5445E+03+	4.5034E+03+	4.1917E+03+	5.2297E+03+	4.3480E+03+	4.3699E+03+	**3.7732E+03-**	4.8254E+03+	4.6153E+03+	4.8913E+03+	3.8684E+03
F24	std	4.1080E+02	2.8889E+02	2.3471E+02	2.8029E+02	1.0460E+02	1.7572E+02	3.3509E+01	3.7850E+02	2.4327E+02	4.8857E+02	5.0686E+02
	mean	7.2688E+03+	5.4694E+03+	5.1671E+03+	6.7668E+03+	6.1624E+03+	4.9296E+03+	4.1928E+03+	6.3353E+03+	4.8438E+03+	6.0168E+03+	**3.8880E+03**
F25	std	3.3547E+03	7.2346E+02	3.9829E+01	1.0842E+02	5.2330E+01	9.2707E+02	6.4201E+01	2.9234E+02	7.2480E+01	1.7349E+02	7.1624E+01
	mean	2.4698E+04+	5.2608E+03+	**3.4050E+03-**	3.9135E+03+	3.5469E+03+	7.2047E+03+	3.6125E+03+	4.6575E+03+	3.6651E+03+	4.6250E+03+	3.5060E+03
F26	std	4.0491E+03	1.9502E+03	2.8954E+03	1.6209E+03	1.0023E+03	4.7049E+03	5.3849E+02	1.7465E+03	6.1990E+03	2.9031E+03	1.5623E+03
	mean	3.2519E+04+	2.1923E+04+	2.4011E+04+	2.7365E+04+	**1.1112E+04-**	2.0345E+04+	1.6319E+04+	2.7695E+04+	1.2985E+04+	2.8574E+04+	1.2411E+04
F27	std	5.1252E+02	3.0597E+02	9.9237E+01	2.3019E+02	7.1126E+01	1.1269E+02	5.0981E+01	3.7836E+02	1.3904E+02	8.6760E+02	5.9827E+02
	mean	6.5495E+03+	4.2398E+03+	3.8411E+03+	4.7224E+03+	3.7581E+03+	3.8120E+03+	3.6785E+03+	5.1597E+03+	4.3553E+03+	5.4160E+03+	**3.5804E+03**
F28	std	4.0875E+03	1.0618E+03	3.8690E+01	1.4075E+02	4.3639E+01	1.1809E+03	6.6739E+01	6.6504E+02	4.5076E+01	6.1087E+02	3.6919E+01
	mean	2.3627E+04+	7.1586E+03+	3.8107E+03+	4.0889E+03+	3.6240E+03+	8.2223E+03+	3.7477E+03+	6.9194E+03+	3.6903E+03+	6.8975E+03+	**3.5923E+03**
F29	std	2.7835E+03	6.6921E+02	8.3572E+02	1.0206E+03	3.9874E+02	9.0782E+02	3.9266E+02	1.7487E+03	1.0213E+03	1.3867E+03	1.2439E+03
	mean	1.5378E+04+	8.6194E+03+	7.5123E+03+	1.0557E+04+	7.9645E+03+	1.1397E+04+	8.8308E+03+	1.3864E+04+	1.0301E+04+	1.3780E+04+	**7.0764E+03**
F30	std	1.5447E+09	2.5726E+07	8.8127E+04	2.3858E+07	2.3857E+07	8.0101E+08	4.1173E+04	3.2222E+08	2.7505E+07	1.4877E+08	2.9654E+06
	mean	2.1940E+09+	3.8676E+07+	5.5207E+06+	6.7254E+07+	5.5303E+07+	3.3008E+08+	1.7836E+05+	4.6860E+08+	4.4888E+07+	3.6049E+08+	**1.6024E+05**
+/≈/-:		29/0/0	28/0/1	28/0/1	29/0/0	24/0/5	28/0/1	27/0/2	29/0/0	25/0/4	29/0/0	~
Avg. rank:		10.79	6.52	4.72	7.31	4.21	7.69	4.62	7.24	3.90	7.21	1.79

Bold values indicate the optimal values, which are consistently used throughout the manuscript.

**Table A6:** Running time of each algorithm on 30-D CEC2017.

	GA	PSO	SSA	HHO	RIME	SOFA	CPO	HO	SIH	MHO	EBOHO
F1	2.2183E−01	1.0161E−01	2.0278E−01	2.3719E−01	1.7449E−01	7.0836E−02	1.4031E−01	4.8621E−01	4.6932E−01	4.7862E−01	3.4293E−01
F3	2.5944E−01	1.4252E−01	2.3390E−01	2.9216E−01	1.8499E−01	1.0886E−01	1.8555E−01	5.2724E−01	4.9798E−01	4.9396E−01	3.5842E−01
F4	2.3104E−01	1.4311E−01	2.7865E−01	3.2398E−01	1.7686E−01	1.0131E−01	1.7832E−01	5.9872E−01	7.2111E−01	6.7756E−01	4.3419E−01
F5	3.1418E−01	2.2612E−01	4.0085E−01	5.5879E−01	2.5785E−01	1.7424E−01	2.5817E−01	6.8811E−01	7.8403E−01	7.4420E−01	5.9320E−01
F6	5.3178E−01	4.0865E−01	6.3597E−01	9.7089E−01	4.4059E−01	3.2815E−01	3.9400E−01	7.2026E−01	8.3202E−01	7.8378E−01	6.4798E−01
F7	3.7855E−01	2.4293E−01	4.2687E−01	5.9772E−01	2.9089E−01	1.9964E−01	2.8758E−01	7.2257E−01	7.2793E−01	6.9078E−01	5.1607E−01
F8	2.8690E−01	2.1834E−01	3.7164E−01	4.9383E−01	2.4141E−01	1.6541E−01	2.3517E−01	6.5558E−01	7.1066E−01	6.7055E−01	5.2066E−01
F9	3.2387E−01	2.2329E−01	3.9943E−01	5.6499E−01	2.6412E−01	1.8484E−01	2.5818E−01	6.9500E−01	7.2814E−01	7.1112E−01	5.5008E−01
F10	4.5001E−01	2.8534E−01	4.7482E−01	6.8196E−01	3.0912E−01	2.4394E−01	2.9947E−01	5.9313E−01	6.3264E−01	6.6845E−01	4.8366E−01
F11	2.5789E−01	1.6483E−01	2.8094E−01	3.6468E−01	1.9909E−01	1.2859E−01	2.0346E−01	5.2780E−01	5.8872E−01	7.5382E−01	6.0749E−01
F12	3.7707E−01	2.4279E−01	4.4617E−01	6.0713E−01	2.9188E−01	1.9368E−01	2.9082E−01	8.1711E−01	8.3955E−01	7.9093E−01	5.6791E−01
F13	3.1172E−01	1.9493E−01	3.5460E−01	4.5715E−01	2.2896E−01	1.4848E−01	2.3803E−01	7.8701E−01	8.3023E−01	7.8771E−01	6.0406E−01
F14	4.2782E−01	2.9745E−01	5.1672E−01	7.4208E−01	3.4249E−01	2.4199E−01	3.4881E−01	8.3455E−01	8.8552E−01	8.3826E−01	6.6285E−01
F15	3.4952E−01	2.1819E−01	4.0468E−01	5.3113E−01	2.6622E−01	1.5953E−01	2.6736E−01	7.5798E−01	7.7540E−01	8.0928E−01	6.0683E−01
F16	3.6666E−01	2.3891E−01	4.3874E−01	5.6860E−01	2.8369E−01	1.8447E−01	2.8509E−01	8.0436E−01	8.3066E−01	7.4054E−01	4.6868E−01
F17	4.6281E−01	3.3250E−01	5.1874E−01	8.7372E−01	3.6018E−01	2.7431E−01	3.6156E−01	7.7136E−01	8.6956E−01	7.7684E−01	6.8760E−01
F18	3.8405E−01	2.4392E−01	4.4959E−01	6.0820E−01	2.9037E−01	1.9556E−01	3.0000E−01	8.2434E−01	8.4946E−01	7.6559E−01	5.9027E−01
F19	1.2937E+00	1.1097E+00	1.5804E+00	2.7558E+00	1.2067E+00	1.0602E+00	1.1534E+00	1.0553E+00	1.0481E+00	1.1018E+00	1.0115E+00
F20	5.1541E−01	3.7385E−01	6.2254E−01	9.5052E−01	4.2306E−01	3.2911E−01	4.2435E−01	8.0599E−01	8.6761E−01	8.1937E−01	6.5242E−01
F21	5.6162E−01	4.2379E−01	6.8242E−01	1.0607E+00	4.9037E−01	3.7242E−01	4.6906E−01	7.6423E−01	8.5106E−01	8.1800E−01	5.5028E−01
F22	5.6035E−01	4.1499E−01	7.0080E−01	9.8002E−01	4.5702E−01	3.6547E−01	4.4858E−01	6.8691E−01	8.0917E−01	8.5477E−01	7.1476E−01
F23	6.7369E−01	5.3140E−01	8.0337E−01	1.1768E+00	5.1556E−01	4.2201E−01	5.0690E−01	8.2641E−01	9.0328E−01	8.5188E−01	7.0971E−01
F24	7.3693E−01	6.0555E−01	9.0141E−01	1.4358E+00	6.3684E−01	5.4197E−01	6.3131E−01	8.9122E−01	9.3589E−01	8.8851E−01	6.6733E−01
F25	5.9724E−01	4.5702E−01	7.6003E−01	1.0660E+00	5.0591E−01	4.0852E−01	4.9740E−01	8.2940E−01	8.8047E−01	8.2589E−01	6.9268E−01
F26	7.5289E−01	6.0351E−01	9.1412E−01	1.4739E+00	6.7403E−01	5.6130E−01	6.5060E−01	8.9989E−01	9.4627E−01	7.8760E−01	7.5035E−01
F27	8.0358E−01	6.5769E−01	9.9114E−01	1.6341E+00	7.0873E−01	6.0931E−01	7.0886E−01	8.7756E−01	8.1737E−01	7.5688E−01	6.5868E−01
F28	6.1776E−01	4.9032E−01	7.2496E−01	1.0996E+00	5.1883E−01	4.3324E−01	5.1942E−01	7.0134E−01	7.8567E−01	8.5083E−01	7.1395E−01
F29	6.3248E−01	4.8172E−01	7.5292E−01	1.1936E+00	5.5929E−01	4.4034E−01	5.2509E−01	8.2500E−01	8.8996E−01	8.5291E−01	7.0888E−01
F30	1.4476E+00	1.1892E+00	1.5370E+00	2.7066E+00	1.2491E+00	1.1467E+00	1.2397E+00	1.0646E+00	1.1578E+00	1.1231E+00	1.0728E+00
Avg.time	5.2167E−01	3.8842E−01	6.1401E−01	9.3129E−01	4.3271E−01	3.3774E−01	4.2436E−01	7.5997E−01	8.0916E−01	7.8323E−01	6.2573E−01

**Table A7:** Running time of each algorithm on 50-D CEC2017.

	GA	PSO	SSA	HHO	RIME	SOFA	CPO	HO	SIH	MHO	EBOHO
F1	4.5962E−01	2.7378E−01	4.7336E−01	5.3297E−01	3.8236E−01	2.4900E−01	4.2020E−01	1.0046E+00	1.0260E+00	1.0456E+00	8.3128E−01
F3	3.8111E−01	2.7126E−01	4.3312E−01	6.1902E−01	3.8148E−01	2.2375E−01	3.8718E−01	1.5266E+00	1.3273E+00	1.5117E+00	1.1647E+00
F4	4.8046E−01	4.0952E−01	6.6406E−01	8.8973E−01	5.1032E−01	2.7937E−01	4.4346E−01	1.4902E+00	1.4765E+00	1.3914E+00	1.1349E+00
F5	6.6834E−01	5.4471E−01	7.9168E−01	1.2131E+00	6.5174E−01	4.2809E−01	5.7069E−01	1.4634E+00	1.4596E+00	1.4768E+00	1.2279E+00
F6	1.0857E+00	1.0127E+00	1.4543E+00	2.2996E+00	1.0451E+00	8.3317E−01	9.7291E−01	1.6343E+00	1.8051E+00	1.9011E+00	1.6958E+00
F7	8.4812E−01	6.8962E−01	1.1048E+00	1.3960E+00	6.8537E−01	5.1032E−01	6.9709E−01	1.9304E+00	1.9981E+00	1.7560E+00	1.4360E+00
F8	8.3745E−01	6.6988E−01	1.0115E+00	1.6078E+00	7.8074E−01	5.0296E−01	6.8149E−01	1.9622E+00	2.0013E+00	1.3947E+00	1.3516E+00
F9	8.5861E−01	6.9396E−01	1.0517E+00	1.6881E+00	7.9544E−01	5.2290E−01	7.2106E−01	1.8918E+00	2.0427E+00	1.4383E+00	1.3118E+00
F10	8.3133E−01	7.2372E−01	1.0184E+00	1.6372E+00	7.8232E−01	5.4268E−01	7.2060E−01	1.7320E+00	1.8373E+00	1.6710E+00	1.3108E+00
F11	6.7194E−01	5.3314E−01	8.6360E−01	1.2282E+00	6.2793E−01	3.7626E−01	5.6208E−01	1.7023E+00	1.4335E+00	1.4174E+00	1.2366E+00
F12	6.8858E−01	5.6559E−01	8.4839E−01	1.3587E+00	7.2539E−01	4.8273E−01	6.4380E−01	1.7153E+00	1.7591E+00	1.5451E+00	1.2894E+00
F13	6.7674E−01	5.4097E−01	8.9686E−01	1.2335E+00	6.3242E−01	3.8844E−01	5.8134E−01	1.6224E+00	1.8279E+00	1.4446E+00	1.2697E+00
F14	9.3781E−01	7.1875E−01	1.0835E+00	1.7117E+00	8.0823E−01	5.7114E−01	7.4347E−01	1.6503E+00	1.7141E+00	1.3227E+00	1.0596E+00
F15	5.6485E−01	3.9412E−01	7.3671E−01	1.0696E+00	5.3736E−01	3.0652E−01	4.7219E−01	1.6290E+00	1.6917E+00	1.4992E+00	1.1913E+00
F16	6.2640E−01	4.8003E−01	7.5243E−01	1.1345E+00	5.8601E−01	3.5357E−01	5.1897E−01	1.5508E+00	1.6880E+00	1.4431E+00	1.0881E+00
F17	9.3211E−01	7.5198E−01	1.0966E+00	1.8025E+00	8.4147E−01	6.3121E−01	8.3374E−01	1.4155E+00	1.5369E+00	1.3265E+00	1.1293E+00
F18	6.2316E−01	4.9001E−01	7.4077E−01	1.1206E+00	5.7137E−01	3.5513E−01	5.4809E−01	1.3286E+00	1.3333E+00	1.0843E+00	1.0243E+00
F19	2.7389E+00	2.5488E+00	3.4160E+00	6.2308E+00	2.7672E+00	2.5304E+00	2.6582E+00	2.0157E+00	2.3173E+00	2.0846E+00	2.0409E+00
F20	9.7376E−01	8.1774E−01	1.1802E+00	2.0504E+00	9.0972E−01	6.8511E−01	8.3493E−01	1.4657E+00	1.5812E+00	1.1998E+00	1.0929E+00
F21	1.1696E+00	1.0412E+00	1.4347E+00	2.3935E+00	1.1060E+00	8.9432E−01	1.0431E+00	1.5096E+00	1.6544E+00	1.4374E+00	1.2670E+00
F22	1.3004E+00	1.1106E+00	1.5792E+00	2.7791E+00	1.2303E+00	1.0135E+00	1.1591E+00	1.5864E+00	1.7168E+00	1.5414E+00	1.3581E+00
F23	1.4067E+00	1.2350E+00	1.7237E+00	2.9591E+00	1.3456E+00	1.2422E+00	1.2616E+00	1.4107E+00	1.6677E+00	1.5451E+00	1.4045E+00
F24	1.6070E+00	1.4258E+00	1.9741E+00	3.4196E+00	1.5169E+00	1.2959E+00	1.4440E+00	1.6397E+00	1.8324E+00	1.6355E+00	1.4949E+00
F25	1.2955E+00	1.1387E+00	1.6194E+00	2.6343E+00	1.2377E+00	1.0121E+00	1.1568E+00	1.5682E+00	1.6437E+00	1.3180E+00	1.2981E+00
F26	1.6761E+00	1.5115E+00	2.0307E+00	3.4013E+00	1.6144E+00	1.3543E+00	1.5028E+00	1.7028E+00	1.8435E+00	1.6568E+00	1.5247E+00
F27	1.8920E+00	1.6830E+00	2.3368E+00	4.0326E+00	1.7947E+00	1.5520E+00	1.7407E+00	1.7426E+00	1.9472E+00	1.7363E+00	1.4448E+00
F28	1.5668E+00	1.4372E+00	1.9631E+00	3.1575E+00	1.5296E+00	1.2698E+00	1.4262E+00	1.6488E+00	1.8294E+00	1.6249E+00	1.4733E+00
F29	1.3216E+00	1.1349E+00	1.6488E+00	2.7833E+00	1.2594E+00	1.0285E+00	1.2164E+00	1.5997E+00	1.7336E+00	1.5414E+00	1.3545E+00
F30	3.1745E+00	2.7629E+00	3.6235E+00	6.3370E+00	2.8836E+00	2.8038E+00	2.9734E+00	2.1579E+00	2.4600E+00	2.2489E+00	2.2523E+00
Avg.time	1.1136E+00	9.5210E−01	1.3639E+00	2.2318E+00	1.0531E+00	8.3584E−01	9.9778E−01	1.6309E+00	1.7305E+00	1.5255E+00	1.3365E+00

## Appendix C. Results of Ablation Experiments in Terms of CEC Benchmarks

Table A8 and Table A9 summarize the results of ablation experiments in terms of CEC2017 benchmarks.

**Table A8:** Results of ablation experiments on 30-D CEC2017.

Func.	HO		HO-Beta		HO-EMO		HO-LIRL		EBOHO	
	Mean	Std	Mean	Std	Mean	Std	Mean	Std	Mean	Std
F1	1.8982E+08	1.1881E+08	1.1147E+08-	9.5010E+07	2.3735E+05-	8.3164E+04	1.7475E+08-	2.3661E+08	**6.0369E+04-**	1.1809E+05
F3	5.0748E+04	7.3728E+03	4.7683E+04-	7.3895E+03	3.5159E+04-	5.2345E+03	5.0153E+04-	6.8989E+03	**1.0922E+04-**	5.8240E+03
F4	6.3438E+02	4.9919E+01	6.2906E+02-	5.0606E+01	5.1424E+02-	3.0121E+01	6.1763E+02-	1.1210E+02	**5.0713E+02-**	4.7084E+01
F5	7.1836E+02	2.2935E+01	7.1353E+02≈	2.8758E+01	**6.9154E+02-**	2.8741E+01	7.1767E+02≈	2.7810E+01	7.0226E+02-	2.2725E+01
F6	6.5556E+02	8.4366E+00	6.5788E+02≈	6.6093E+00	6.5261E+02≈	8.2567E+00	6.5673E+02≈	8.8380E+00	**6.1111E+02-**	5.7738E+00
F7	1.1269E+03	7.2758E+01	1.1076E+03-	4.5445E+01	1.1332E+03+	7.9952E+01	1.0949E+03-	6.0224E+01	**9.1078E+02-**	4.0058E+01
F8	9.6086E+02	2.5593E+01	9.5212E+02-	2.5260E+01	9.2983E+02-	2.8057E+01	9.3315E+02-	2.5100E+01	**8.8420E+02-**	1.4170E+01
F9	5.7249E+03	1.1056E+03	5.8138E+03+	6.4428E+02	5.4521E+03-	1.0928E+03	5.3083E+03-	6.9417E+02	**4.6450E+03-**	1.0930E+03
F10	5.3332E+03	5.6491E+02	5.3116E+03-	6.0229E+02	5.2639E+03-	5.8985E+02	5.2117E+03-	5.3015E+02	**5.1184E+03-**	6.5921E+02
F11	1.7173E+03	1.6587E+02	1.6833E+03-	1.2616E+02	**1.2556E+03-**	5.9700E+01	1.5763E+03-	9.3501E+01	1.2577E+03-	4.9756E+01
F12	8.1722E+07	3.3247E+07	7.9250E+07-	5.4356E+07	2.9674E+06-	2.4247E+06	6.5368E+07-	4.2819E+07	**1.4117E+06-**	9.4939E+06
F13	1.2408E+05	7.9418E+04	1.1830E+05-	9.1291E+04	8.6822E+04-	5.2121E+04	1.3719E+05+	7.5820E+04	**2.0095E+04-**	2.4161E+04
F14	2.8075E+05	3.5532E+05	3.0792E+05+	2.3052E+05	2.9990E+04-	2.0016E+04	2.2393E+05-	2.0621E+05	**3.1104E+03-**	6.3640E+04
F15	2.9038E+04	1.7564E+04	2.8666E+04-	3.2159E+04	1.5034E+04-	1.0535E+04	2.3721E+04-	1.6984E+04	**1.1222E+04-**	1.6150E+04
F16	3.3372E+03	2.9456E+02	3.2961E+03-	3.8189E+02	3.1532E+03-	2.1848E+02	3.3020E+03-	4.8043E+02	**2.2574E+03-**	4.6886E+02
F17	2.4435E+03	2.0770E+02	2.3222E+03-	1.4209E+02	2.2720E+03-	1.5485E+02	2.5266E+03+	1.5835E+02	**2.0945E+03-**	1.7273E+02
F18	7.3029E+05	7.4319E+05	6.3166E+05-	9.1806E+05	5.6017E+05-	1.0768E+06	6.4053E+05-	5.5417E+05	**9.6647E+04-**	1.0198E+05
F19	2.9936E+06	1.9193E+06	2.5682E+06-	1.0044E+06	**1.9095E+04-**	1.7087E+04	2.6657E+06-	2.2554E+06	1.9250E+04-	3.1880E+04
F20	2.4673E+03	1.0182E+02	2.4247E+03-	7.7527E+01	2.4133E+03-	1.1957E+02	2.4236E+03-	8.8686E+01	**2.3206E+03-**	9.0161E+01
F21	2.5213E+03	4.3664E+01	2.5067E+03-	4.0980E+01	2.4574E+03-	4.6043E+01	2.5196E+03≈	5.1830E+01	**2.2140E+03-**	2.7988E+01
F22	3.8044E+03	1.7382E+03	4.0219E+03+	2.2918E+03	3.1497E+03-	1.7169E+03	5.1830E+03+	2.3453E+03	**2.3118E+03-**	4.1423E+00
F23	2.9645E+03	8.6147E+01	2.9940E+03+	1.0947E+02	2.9676E+03≈	5.8439E+01	2.9771E+03+	1.1457E+02	**2.8092E+03-**	1.1145E+02
F24	3.1235E+03	8.6219E+01	3.1229E+03≈	8.4117E+01	3.1025E+03-	6.8053E+01	3.1469E+03+	8.8433E+01	**2.8925E+03-**	9.4172E+01
F25	3.0169E+03	2.1779E+01	3.0043E+03-	2.3371E+01	2.9337E+03-	1.8688E+01	3.0032E+03-	4.7576E+01	**2.8303E+03-**	3.2670E+01
F26	6.9250E+03	1.6309E+03	6.3957E+03-	1.0211E+03	7.0502E+03+	8.7863E+02	6.6505E+03-	1.0127E+03	**3.1422E+03-**	9.4792E+02
F27	3.4704E+03	1.1772E+02	3.4267E+03-	4.9747E+01	3.4170E+03-	1.6687E+02	3.4011E+03-	8.8255E+01	**3.2850E+03-**	1.4982E+02
F28	3.3820E+03	6.4643E+01	3.3950E+03+	5.3761E+01	**3.2471E+03-**	2.2447E+01	3.4174E+03+	1.0395E+02	3.2663E+03-	2.9612E+01
F29	4.7933E+03	4.0268E+02	4.6676E+03-	4.2216E+02	4.5987E+03-	3.3282E+02	4.6584E+03-	4.3626E+02	**3.9845E+03-**	2.5263E+02
F30	2.1049E+07	1.4895E+07	2.1009E+07-	1.9442E+07	4.0608E+05-	3.4286E+05	1.5681E+07-	1.4854E+07	**1.9859E+04-**	5.0070E+05
+/≈/-:	-		4/3/22		2/2/25		6/3/20		0/0/29	
Avg.ranks:	4.45		3.69		2.20		3.52		1.14	

Bold values indicate the optimal values, which are consistently used throughout the manuscript.

**Table A9:** Results of ablation experiments on 50-D CEC2017.

Func.	HO		HO-Beta		HO-EMO		HO-LIRL		EBOHO	
	Mean	Std	Mean	Std	Mean	Std	Mean	Std	Mean	Std
F1	2.1098E+09	1.0946E+09	2.0292E+09-	1.1689E+09	3.9722E+05-	1.4907E+05	1.5264E+09-	9.3700E+08	**3.6977E+05-**	8.7197E+05
F3	1.0624E+05	1.3159E+04	1.0713E+05+	7.7005E+03	6.9996E+04-	1.1896E+04	1.0345E+05-	1.5673E+04	**3.4102E+04-**	1.2502E+04
F4	1.2477E+03	1.7632E+02	1.1043E+03-	2.1632E+02	**5.7929E+02-**	5.6274E+01	1.0074E+03-	1.6547E+02	6.2233E+02-	4.3429E+01
F5	8.9063E+02	5.0901E+01	8.5478E+02-	3.2146E+01	**8.3220E+02-**	2.2876E+01	8.6253E+02-	3.4937E+01	8.5077E+02-	2.0901E+01
F6	6.6872E+02	5.6443E+00	6.6621E+02≈	6.2672E+00	**6.6278E+02-**	3.4571E+00	6.6921E+02≈	5.3526E+00	6.6472E+02≈	5.4184E+00
F7	1.6399E+03	7.7513E+01	1.6305E+03-	8.9388E+01	1.6106E+03-	7.8070E+01	1.6124E+03-	1.5386E+02	**1.0128E+03-**	1.1601E+02
F8	1.1295E+03	4.2579E+01	1.1472E+03+	5.9026E+01	1.0942E+03-	4.2570E+01	1.1199E+03-	3.8010E+01	**9.8739E+02-**	1.9134E+01
F9	1.7237E+04	1.8820E+03	1.8348E+04+	2.2350E+03	1.6650E+04-	2.2776E+03	1.5496E+04-	2.3578E+03	**2.5031E+03-**	3.5446E+03
F10	8.3898E+03	1.1707E+03	8.1294E+03-	7.4152E+02	8.1087E+03-	6.7449E+02	9.1038E+03+	1.0069E+03	**7.5299E+03-**	9.7283E+02
F11	2.5195E+03	4.9898E+02	2.5097E+03-	2.9487E+02	1.4154E+03-	9.8381E+01	2.3835E+03-	1.6098E+02	**1.4139E+03-**	5.6386E+01
F12	3.8017E+08	2.2886E+08	3.0073E+08-	3.1612E+08	3.6135E+07-	2.1278E+07	3.6548E+08-	3.0972E+08	**9.8760E+06-**	4.9763E+07
F13	4.5778E+06	1.2482E+07	1.9836E+05-	1.3873E+05	**8.3918E+04-**	2.7751E+04	2.1455E+05-	1.3523E+05	8.8808E+04-	3.8577E+04
F14	1.2271E+06	7.0079E+05	1.1223E+06-	8.2424E+05	5.1294E+05-	6.1946E+05	1.1970E+06-	1.9778E+06	**4.6482E+04-**	3.8520E+05
F15	4.8871E+04	3.7237E+04	3.8347E+04-	3.8299E+04	2.6964E+04-	1.2742E+04	3.1178E+04-	7.0105E+04	**2.5300E+04-**	1.8945E+04
F16	4.7051E+03	7.7144E+02	4.4842E+03-	4.6980E+02	4.0013E+03-	5.1922E+02	4.5614E+03-	4.8397E+02	**3.9044E+03-**	6.3559E+02
F17	3.6978E+03	3.2642E+02	3.7494E+03+	4.2424E+02	3.6910E+03-	4.7973E+02	3.5002E+03-	4.6578E+02	**3.1152E+03-**	3.6713E+02
F18	3.3938E+06	2.6089E+06	3.2944E+06-	1.8476E+06	1.7532E+06-	1.3215E+06	3.1804E+06-	2.3907E+06	**5.8216E+05-**	7.4233E+05
F19	4.4081E+06	5.4043E+06	7.7372E+06+	6.4571E+06	3.4006E+04-	3.6073E+04	3.2671E+06-	5.4806E+06	**3.3830E+04-**	1.9563E+04
F20	3.1104E+03	2.1444E+02	3.1039E+03-	2.3098E+02	3.0373E+03-	2.0253E+02	3.0498E+03-	2.5614E+02	**3.0253E+03-**	1.7163E+02
F21	2.7387E+03	6.3394E+01	2.7294E+03-	5.6638E+01	2.6966E+03-	7.1621E+01	2.7284E+03-	7.6365E+01	**2.3342E+03-**	6.6870E+01
F22	1.0382E+04	8.4201E+02	1.0132E+04-	9.9555E+02	1.0724E+04+	1.3721E+03	1.0158E+04-	9.5304E+02	**9.1833E+03-**	1.1244E+03
F23	3.4069E+03	9.6413E+01	3.3759E+03-	1.4253E+02	3.3457E+03-	1.6539E+02	3.4638E+03+	1.7369E+02	**3.0636E+03-**	1.4310E+02
F24	3.7322E+03	1.7843E+02	3.6733E+03-	1.4717E+02	3.6561E+03-	1.6230E+02	3.7365E+03+	2.0507E+02	**3.1176E+03-**	1.7378E+02
F25	3.6068E+03	1.8924E+02	3.5167E+03-	2.0192E+02	**3.1162E+03-**	2.4093E+01	3.4737E+03-	1.4375E+02	3.1245E+03-	2.7962E+01
F26	1.1120E+04	1.3611E+03	1.1071E+04-	2.1359E+03	1.0906E+04-	2.7779E+03	1.1007E+04-	3.2101E+03	**4.4358E+03-**	1.3138E+03
F27	4.1703E+03	2.5620E+02	4.1564E+03-	2.9383E+02	4.1102E+03-	3.2386E+02	4.1160E+03-	2.3027E+02	**3.3987E+03-**	4.6162E+02
F28	4.3322E+03	2.6587E+02	4.2599E+03-	1.9589E+02	**3.4034E+03-**	2.7051E+01	4.1895E+03-	1.8620E+02	3.4134E+03-	4.0566E+01
F29	7.6092E+03	5.5228E+02	7.5231E+03-	1.1040E+03	6.2545E+03-	8.2091E+02	8.1320E+03+	1.0462E+03	**4.8692E+03-**	5.5798E+02
F30	2.1608E+08	7.5207E+07	2.0713E+08-	3.6552E+07	2.0703E+07-	7.9099E+06	1.9624E+08-	8.0338E+07	**1.4591E+06-**	1.0786E+07
+/≈/-:	-		5/1/23		1/0/28		4/1/24		0/1/28	
Avg.ranks:	4.62		3.75		1.97		3.45		1.21	

Bold values indicate the optimal values, which are consistently used throughout the manuscript.

## Appendix D. Basic Information on Engineering Problems

### Appendix D.1. Pressure Vessel Design Problem (PVDP)

The mathematical model of the Pressure Vessel Design Problem (PVDP) is formulated in Equation (A1) and demonstrated in Figure A1.

(A1) $$\begin{array}{l} \min\begin{array}{c} \;\;\;\;\;\;f(X) \end{array}=0.6224x_{1}x_{3}x_{4}+1.7781x_{2}x_{3}^{2}+3.1661x_{1}^{2}x_{4}+19.84x_{1}^{2}x_{3}\\ {\begin{array}{c} \begin{array}{cc} \;\;\;\;s.t. & g \end{array} \end{array}}_{1}(X)=-x_{1}+0.0193x_{3}\leq 0\\ \;\;\;\;\;\;\;\;\;\;\;\;\;\;g_{2}(X)=-x_{2}+0.00954x_{3}\leq 0\\ \;\;\;\;\;\;\;\;\;\;\;\;\;\;g_{3}(X)=-\pi x_{3}^{2}x_{4}-\frac{4}{3}\pi x_{3}^{3}+1296000\leq 0\\ \;\;\;\;\;\;\;\;\;\;\;\;\;\;g_{4}(X)=x_{4}-240\leq 0\\ \begin{array}{cc} where & \end{array}\;\;\;0\leq x_{1},x_{2}\leq 99\;\;\;\;\begin{array}{c} 10\leq \end{array}x_{3},x_{4}\leq 200 \end{array}$$

### Appendix D.2. Tubular Column Design Problem (TCDP)

The mathematical model of the Tubular Column Design Problem (TCDP) is formulated in Equation (A2) and demonstrated in Figure A2.

(A2) $$\begin{array}{l} \min\begin{array}{c} \;\;\;\;\;\;f(X) \end{array}=9.8x_{1}x_{2}+2x_{1}\\ \begin{array}{cc} \;\;\;\;\;s.t. & g_{1} \end{array}(X)=\frac{P}{\pi x_{1}x_{2}\sigma_{y}}-1\leq 0\\ \begin{array}{c} \;\;\;\;\;\;\;\;\;\;\;\;\;\;g_{2} \end{array}(X)=\frac{8PL^{2}}{\pi^{3}Ex_{1}x_{2}(x_{1}^{2}+x_{2}^{2})}-1\leq 0\\ \begin{array}{c} \;\;\;\;\;where \end{array}\begin{array}{c} \;\;\;\;\;\;2 \end{array}\leq x_{1}\leq 14,0.2\leq x_{2}\leq 0.8 \end{array}$$

### Appendix D.3. Three-Bar Truss Design Problem (TBTDP)

The mathematical model of the Three-Bar Truss Design Problem (TBTDP) is formulated in Equation (A3) and demonstrated in Figure A3.

(A3) $$\begin{array}{l} \min\begin{array}{c} \;\;\;\;\;\;\;f(X) \end{array}=(2\sqrt{2}x_{1}+x_{2})\cdot l\\ \begin{array}{c} \begin{array}{cc} \;\;\;\;\;s.t. & g_{1}(X) \end{array} \end{array}=\frac{\sqrt{2}x_{1}+x_{2}}{\sqrt{2}x_{1}^{2}+2x_{1}x_{2}}p-\sigma \leq 0\\ \begin{array}{c} \begin{array}{c} {\;\;\;\;\;\;\;\;\;\;\;\;\;\;g}_{2}(X) \end{array} \end{array}=\frac{x_{2}}{\sqrt{2}x_{1}^{2}+2x_{1}x_{2}}p-\sigma \leq 0\\ \begin{array}{c} \begin{array}{c} {\;\;\;\;\;\;\;\;\;\;\;\;\;\;g}_{3}(X) \end{array} \end{array}=\frac{1}{\sqrt{2}x_{2}+x_{1}}p-\sigma \leq 0\\ \begin{array}{c} \begin{array}{c} \;\;\;\;\;\;\;\;\;\;\;\;\;\;l= \end{array} \end{array}100\mathrm{cm},p=2\mathrm{kN}/{\mathrm{cm}}^{3},\sigma =2\mathrm{kN}/{\mathrm{cm}}^{3}\\ \begin{array}{c} \begin{array}{cc} \;\;\;\;\;where & \;\;\;\;\;\;0\leq x_{1},x_{2}\leq 1 \end{array} \end{array} \end{array}$$

### Appendix D.4. Piston Lever Design Problem (PLDP)

The mathematical model of the Piston Lever Design Problem (PLDP) is formulated in Equation (A4) and demonstrated in Figure A4.

(A4) $$\begin{array}{l} \min\begin{array}{cc} & \;\;\;\;\;\;f(X) \end{array}=\frac{1}{4}\pi x_{3}^{2}(L_{2}-L_{1})\\ \begin{array}{c} \begin{array}{cc} s.t. & \;g_{1}(X) \end{array} \end{array}=QL\cos(\theta )-R\times F\leq 0\\ \begin{array}{c} \begin{array}{cc} & {\;g}_{2}(X) \end{array} \end{array}=Q(L-x_{4})-M_{\max}\leq 0\\ \begin{array}{c} \begin{array}{cc} & {\;g}_{3}(X) \end{array} \end{array}=1.2(L_{2}-L_{1})-L_{1}\leq 0\\ \begin{array}{c} \begin{array}{cc} & {\;g}_{4}(X) \end{array} \end{array}=\frac{x_{3}}{2}-x_{2}\leq 0\\ \begin{array}{c} \begin{array}{cc} \;\;\;\;\;\;\;\;where & \;R=\frac{\left|-x_{4}(x_{4}\sin\theta +x_{1})+x_{1}(x_{2}-x_{4}\cos\theta )\right|}{\sqrt{{\left(x_{4}-x_{2}\right)}^{2}+x_{1}^{2}}} \end{array} \end{array}\\ \begin{array}{c} F= \end{array}\frac{\pi Px_{3}^{2}}{4},L_{1}=\sqrt{{\left(x_{4}-x_{2}\right)}^{2}+x_{1}^{2}},L_{2}=\sqrt{{\left(x_{4}\sin\theta +x_{1}\right)}^{2}+{\left(x_{2}-x_{4}\cos\theta \right)}^{2}}\\ \begin{array}{c} \theta =45^{{}^{\circ}} \end{array},Q=10000\mathrm{lbs},L=240\mathrm{in},M_{\max}=1.8\times 10^{6}\begin{array}{cc} \mathrm{lbs} & \mathrm{in}, \end{array}P=1500\mathrm{psi}\\ \begin{array}{c} 0.05\leq x_{1},x_{2},x_{4}\leq 500,0.05\leq x_{3}\leq 120 \end{array} \end{array}$$
